# Topological assessment of metabolic networks reveals evolutionary information

**DOI:** 10.1038/s41598-018-34163-7

**Published:** 2018-10-29

**Authors:** Jeaneth Machicao, Humberto A. Filho, Daniel J. G. Lahr, Marcos Buckeridge, Odemir M. Bruno

**Affiliations:** 1 São Carlos Institute of Physics, University of São Paulo, São Carlos, SP PO Box 369, 13560-970 Brazil; 2 Institute of Biosciences, University of São Paulo, São Paulo, SP Brazil

## Abstract

Evolutionary information was inferred from the topology of metabolic networks corresponding to 17 plant species belonging to major plant lineages *Chlorophytes*, *Bryophytes*, *Lycophytes* and *Angiosperms*. The plant metabolic networks were built using the substrate-product network modeling based on the metabolic reactions available on the PlantCyc database (version 9.5), from which their local topological properties such as degree, in-degree, out-degree, clustering coefficient, hub-score, authority-score, local efficiency, betweenness and eigencentrality were measured. The topological measurements corresponding to each metabolite within the networks were considered as a set of metabolic characters to compound a feature vector representing each plant. Our results revealed that some local topological characters are able to discern among plant kinships, since similar phylogenies were found when comparing dendrograms obtained by topological metrics to the one obtained by DNA sequences of chloroplast genes. Furthermore, we also found that even a smaller number of metabolic characters is able to separate among major clades with high bootstrap support (BS > 95), while for some suborders a bigger content has been required.

## Introduction

Phylogenetic studies of plants were initiated by morphological observations related to their reproductive organs^1^. The molecular biology and recombinant DNA technology enabled the evaluation of the evolutionary relationships by comparison of highly conserved DNA primary sequence of plastid genes, such as the successfully established phylogenetic relationships between plants by correctly discerning the ancestry relations^2^.

Many works have used the metabolic network content and its topology as an alternative tool to assess the phylogenetic relationships^3–14^. Early approaches to construct phylogenetic trees were applied using, for instances, graph matching comparison algorithm^4^, graph kernel method^5^ and graph-based decomposition algorithm^6^ to organisms such as *archaea*, *eukaryota*, and *bacteria*. Other authors have traced phylogenetic distances between metabolic networks using network’s global properties, for instances, Zhu & Qin^7^ used higher-level topological properties (network indices, degree distribution measures, and motif profile measure), while others works have explored the spectral distribution (Laplacian spectrum) in different approaches^8–10^. Further attempts have used alternative models such as NIPs (network of interacting pathways)^11^, from which several network-descriptors are extracted in order to predict phylogenetic distances within these three living beings groups. Besides that, protein-protein interaction networks were explored as well based on the identification of modular network components from each network^12^, and graph alignment for the cross-species analysis applied on *Homo sapiens* and *Mus musculus* species^13^.

Other efforts have brought progress to better understand the evolution of plants by means of their molecular networks. In that regard, Chae *et al*.^14^ have shown phylogenetic similarities based on the comparison of the content of reactions among plant species. However, less attention has been put to the local topological properties of metabolic networks. It could be applied to explore the separation of organisms belonging to a specific kingdom such as *Viridiplantae* in their respective phyla, classes, orders and so on.

Indeed, exploring the scale-free properties of metabolic networks, similarly to how non-biological networks are studied^15–17^, has brought great advances. In general, the existence of hubs is one of the most evident characteristics of scale-free networks, which is corroborated by the correlation between the average shortest path length and the average clustering coefficient^17^. The existence of hubs is a direct consequence of the fact that many links tend to be established on nodes with high connectivity. This scale-free property on metabolic networks indicates that the probability of finding a highly connected node is conserved among living beings^18^. Therefore, the analysis of local topological properties from metabolic networks based on most representative measures such as the degree distribution, clustering coefficient, hub score and many other^18^, may allow finding evolutionary information that is hidden within their complex connections.

In this work, we propose a method to extract the topological metrics of plant metabolic networks, in order to compare it between different plants, to obtain phylogenetic signal. We considered 17 plants, namely: *Brachypodium distachyon* (BD), *Hordeum vulgare* (HV), *Oryza sativa japonica* (OSJ), *Panicum virgatum* (PV), *Setaria italica* (SI), *Sorghum bicolor* (SB), *Zea mays* (ZM); *Arabidopsis thaliana* (AT), *Brassica rapa pekinensis* (BRP), *Carica papaya* (CP), *Glycine max* (GM), *Manihot esculenta* (ME), *Populus trichocarpa* (PT), *Vitis vinifera* (VV); *Selaginella moellendorffii* (SM); *Physcomitrella patens* (PP) and *Chlamydomonas reinhardtii* (CR); whose metabolic pathways, catalytic enzymes and metabolites are available at PlantCyc database^19^.

Features were extracted using a representative set of local topological measurements from the 17 metabolic networks. These features were then submitted to hierarchical clustering methods which provided means to compare it with phylogenetic data obtained from the plastid DNA primary sequence analysis. The results showed that our method manages to obtain information about kinship relationships, mainly in nodes *Chlorophyta*, *Lycophyta*, *Bryophyta* and *Angiosperm* that is divided into monocotyledons and dicotyledons. We observed that the local topological measure called hub-score provided quantitative parameters that were capable to group correctly the species studied within their respective clades.

## Results

### Plant metabolic networks topology

The plant metabolic networks were modeled based on the metabolic reactions corresponding to the 17 plants of the PlantCyc^19^ (version 9.5) (see Supplementary Dataset S1–S3). A summary of measurements extracted from these metabolic networks is shown in Supplementary Information S1. The size of the networks were quantified in terms of the number of reactions and metabolites. For instance, the smallest and highest network contains 2433 and 3546 metabolites corresponding to *Chlamydomonas reinhardtii* and *Arabidopsis thaliana col*, while these same plants contain 2208 and 3424 reactions, respectively. We found 1880 metabolites and 1149 metabolic reactions in common between all the 17 plants studied here. These 1880 metabolites represent 64.3% of information of the average number of metabolites, i.e. 2923, are present in the 17 networks, while 1149 common reactions represent 42.6% of information of the average number of the metabolic reactions, i.e. 2696 (see Supplementary Dataset S4–S6).

**Figure 1:**
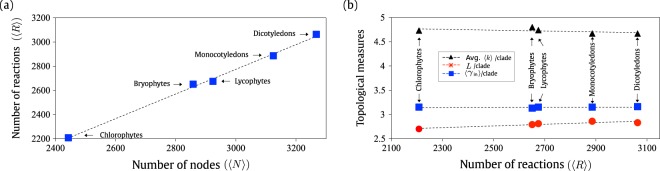
(**a**) Average number of reactions (〈*R*〉) versus the average number of metabolites (〈*N*〉) per plant clade. The plant clades: *Dicotyledons*, *Monocotyledons*, *Lycophytes*, *Bryophytes* and *Chlorophytes* are listed according with their average number of nodes and reactions. (**b**) The average number of plant metabolic reactions in function of the average values of three topological measures namely, mean degree 〈$\bar{k}$〉 (triangle), average path length *L* (circle) and the incoming power-law exponent *γ*_in_ (square), per each plant clade.

We analyzed the size of the metabolic networks and its dispersion throughout the taxonomic classes. Fig. 1a depicts the correlation between the average number of nodes (〈*N*〉) in terms of the average number of reactions (〈*R*〉) present on each major plant clade. This plot shows that the number of metabolites varies linearly with the number of reactions across the plant clades. This data suggests that the metabolic reactions have been accumulated along the evolution of the species as already verified in earlier works^14^.

Global topological measurements (such as mean degree 〈$\bar{k}$〉, average path length 〈*L*〉, the incoming and outgoing power-law exponent *γ*_in_ and *γ*_out_) were calculated for each of the five major clades considered here, i.e. *Chlorophyta*, *Lycophyta*, *Bryophyta*, monocotyledons and dicotyledons. Figure 1b depicts the measurements in function of the average number of reactions (〈*R*〉) per plant clade. Observe that only the the in-going power-law exponent is showed in Fig. 1b, since both the incoming and outgoing power-law exponents do not vary between the plant clades. These results reinforce the fact that plant metabolic networks are scale-free networks, as stated by Jeong *et al*.^18^. The average path length 〈*L*〉, varies slightly with the number of reactions, as it was observed in earlier works^20^. Moreover, as expected, a small slope can be noticed in the curve of the average degree 〈*k*〉 in function of the average number of reactions (〈*R*〉) per plant clade, hence the number of nodes (metabolites) increases from chlorophytes to dicots (Supplementary Information S1).

### Phylogenetic information at local topology of the metabolic network

Nine local topological measurements were used independently to assembly feature vectors from the plant metabolic networks studied here, namely the degree (*k*_*i*_), in-degree (${k}_{i}^{{\rm{in}}}$), out-degree $({k}_{i}^{{\rm{out}}})$, hub-score (H(*v*_*i*_)), local clustering coefficient (C_*i*_), authority-score (A(*v*_*i*_)), local efficiency (F_i_), betweenness (B_*i*_) and eigencentrality (E_*i*_). We used these former topological metrics since they are related to the connectivity, distance-based and centralities measurements. A feature vector *ϕ*_*P*_ was composed using the topological measures of *m* = 1880 metabolites corresponding to the size of the *common-metabolites-set* (see Material and methods section).

**Figure 2:**
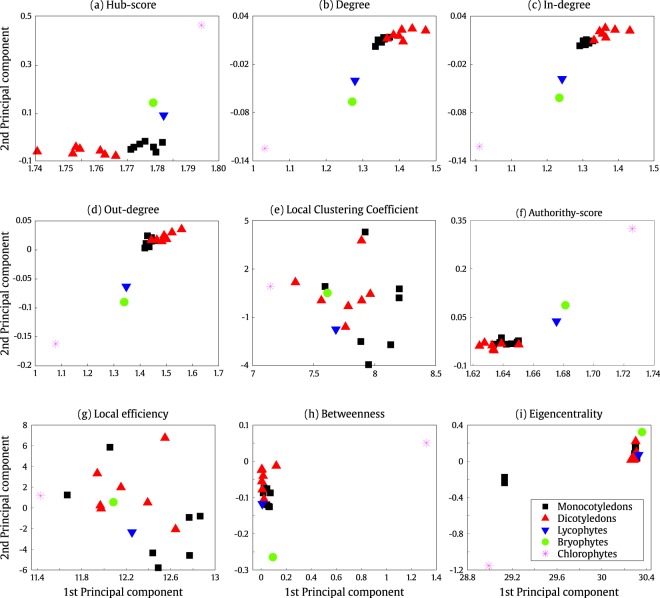
Scatter plot of the two principal components of the feature vector corresponding to the topological networks measures, namely (**a**) hub-score, (**b**) degree, (**c**) in-degree, (**d**) out-degree, (**e**) local clustering coefficient, (**f**) authority-score, (**g**) local efficiency, (**h**) betweenness and (**i**) eigencentrality.

Figure 2 shows a scatter plot of the first and second principal component analysis (PCA) applied to each of the nine feature vectors. We can observe that the hub-score plot (Fig. 2a) is one of the measurements that best clusterizes plants belonging to the same clade, i.e. being able to distinguish between major clades, while the other five measures (degree, in-degree, out-degree, authorithy-score and betweenness in Fig. 2b–d,f,h) barely distinguish between Monocotyledons and Dicotyledons. Conversely, the local clustering coefficient and the local efficiency measures are not able to group plants of same clades (Fig. 2e,g,i).

### Phylogenetic hierarchy inferred by the local topology

We analyzed the distance matrix obtained by the hub-score metric (Supplementary Information S2), where each matrix element represents the Euclidean distance between each pair of plants among the 17 studied. In a similar way, a distance matrix was calculated for the degree, in-degree, out-degree, local clustering coefficient, authority-score, local efficiency, betweenness and eigencentrality, respectively (Supplementary Information S3–S10).

The hierarchical clustering method UPGMA (Unweighted Pair Group Method with Arithmetic mean)^21^ was employed in order to depict a dendrogram. Then the cophenetic correlation coefficient (CPCC) was used to measure the correlation between the distance matrices obtained by the topological measurements and the phylogenetic distance matrix obtained by the plastid gene sequence (Supplementary Information S20).

**Figure 3:**
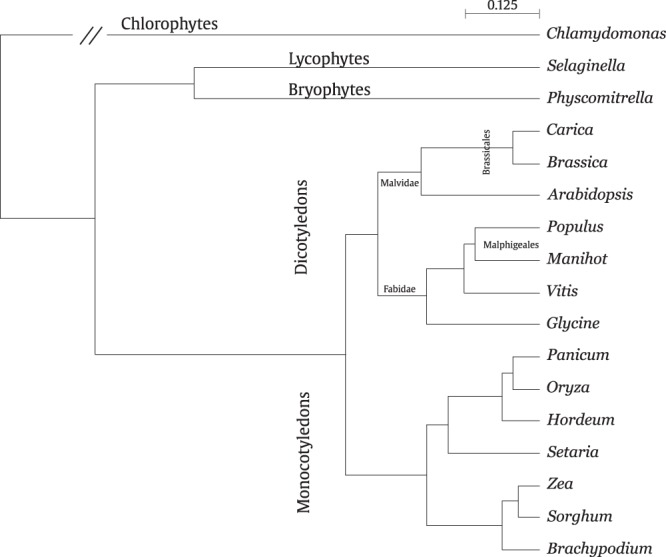
The hub-score dendrogram corresponding to the highest cophenetic coefficient correlation (CPCC = 0.88) respect to the phylogenetic distance matrix based on multiple alignment of 78 plastid gene sequences. The dash containing the number 0.125 shows the distance scale unit of the dendrogram.

The dendrogram obtained by the hub-score metrics shown in Fig. 3 achieved CPCC = 0.88, which demonstrates a taxonomical discernment of the five major clades. The dicots species used in this study belongs to *Rosidae* clade; Inside this clade, the species *Populus trichocarpa*, *Manihot esculenta* and *Glycine max* were correctly assigned as *Fabidae* subclade; and *Carica papaya*, *Brassica rapa pekinensis* and *Arabidopsis thaliana* into the *Malvidae* subclade. It was also found a correct assignment of species within the *Fabidae* and *Malvidae* subclades. The species *Populus trichocarpa*, and *Manihot esculenta* were correctly assigned at *Malpighiales* order; and the species *Brassica rapa pekinensis* and *Arabidopsis thaliana* into *Brassicales* order. These results show the discernment of the method inside the dicots, on the other hand, the same was not observed inside the monocots clade.

**Figure 4:**
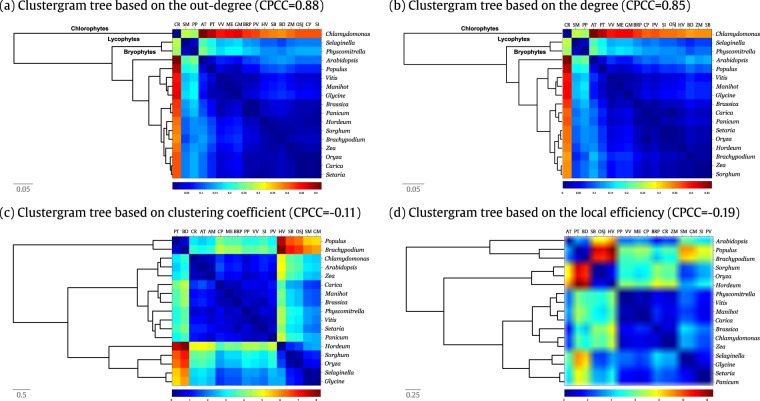
Clustergram of the 17 plant metabolic networks based on local topological metrics, namely (**a**) out-degree, (**b**) degree, (**c**) local clustering coefficient and (**d**) local efficiency. The dash containing numbers (0.05, 0.5 and 0.25) shows the distance scale unit of the dendrogram. The heat map showed at the right of each dendrogram indicates the clustering grouping similarity.

In a similar manner, the other metrics were analyzed as well. The CPCCs obtained by the degree, in-degree, out-degree, local clustering coefficient, authority-score, local efficiency, betweenness and eigencentrality metrics were 0.85, 0.85, 0.88, −0.11, 0.71, −0.19, 0.72 and 0.29, respectively. We show in Fig. 4, the clustergrams corresponding to the out-degree and degree metrics, which obtained the second and third largest CPCC, and the local clustering coefficient and local efficiency metrics, which obtained the lowest values, respectively. The other clustergrams are found at the Supplementary Information S21. The heat map showed at the right of each dendrogram indicates the clustering grouping. As we can observe that the dendrograms corresponding to the degree, in-degree, out-degree, authority-score and betweenness obtained also high CPCCs, contrarily, the local clustering coefficient, local efficiency and eigencentrality has completely failed. Notice that the clustergrams with larger CPCC distinguishes among four clades (*Chlorophytes*, *Bryophytes*, *Lycophytes*, and *Angiosperms*). These metrics introduce attribution errors only within the group of angiosperms with ramifications including both monocotyledonous and dicotyledonous plants at the same branch.

### Validation of the evolutionary information from local topology

We obtained consistent information regarding the discrimination between major clades, as shown in Figs 3 and 4a,b, using the *common-metabolites-set*, however, this dataset excludes those specific metabolites of each plant, which need to be explored as well. Thus, we also analyzed the complete number of metabolites present in all the plants studied here. Therefore, we also considered the *full-metabolites-set*, which contains *m* = 4583 metabolites. We repeated all of the experiments shown previously and we have obtained very similar results, i.e. a high discernment among major clades defined by its higher CPCCs, and more particularly, when using the hub-score metric. The distances matrices corresponding to the nine topological metrics using the *full-metabolites-set* are given in the Supplementary Information S11–S19, while the corresponding scatter plots and clustergrams are in the Supplementary Information S22.

**Figure 5:**
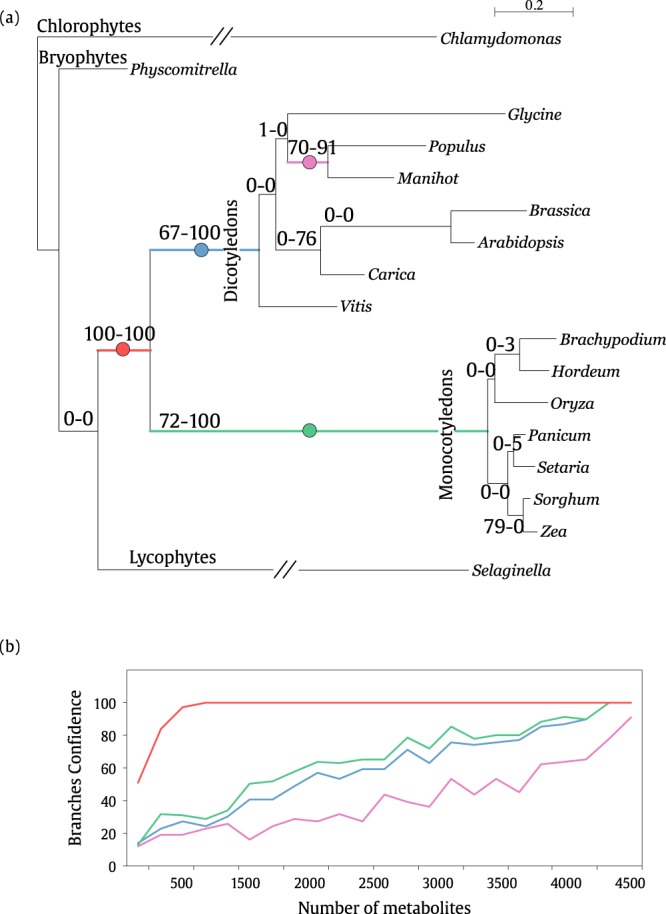
(**a**) The DNA phylogenetic tree corresponding to the highest cophenetic coefficient correlation (CPCC = 0.88) respect to the phylogenetic distance matrix based on multiple alignments of 78 plastid gene sequences (Supplementary Information S20). The dash containing the number 0.125 shows the distance scale unit of the dendrogram. The numbers indicate support from 100 bootstrap rounds based a randomization from the hub-score vector with a range between 100–4500 characters. The colored circles at each branch represent the assignment of branch support corresponding to the curves in (**b**). (**b**) The support of each branch of the hub-score tree based at bootstrap rounds according to the hubs-core feature vector enlargement between 100 and 4500 vector elements (metabolites).

In order to validate the method, we compared the dendrograms obtained from the hub-score metric with the phylogenetic reconstruction regarding the 17 plants, i.e. a “guide tree” obtained from the DNA alignment (see Material and methods section and Supplementary Information S20). The DNA phylogenetic reconstruction is shown in Fig. 5a. The branches shown two confidence values obtained from the bootstrap applied to the hub-score feature vector for both the *common-metabolites-set* and the *full-metabolites-set*, left and right, respectively. The bootstrap values correspond to 100 random replicas obtained by the hub-score dendrogram with feature vectors containing both dataset, i.e. *m* = 1880 and *m* = 4583 characters, using current RAxML (Randomized Axelerated Maximum Likelihood, version 8.2.11)^22^.

One can observe in Fig. 5a that some of the branches (colored lines) shown high taxonomic discernment of monocots and dicots (BS 100 and 100). Moving within the dicots subclade, it can be observed two relatively high confidence support values on branches enclosing CP, AT and BRP (BS 67-100) and PT and ME (BS 70-91). In contrast, the same feature vector provided lower BS for the rest of branches. Moving back to the monocots subclades, the species BD, HV, and OSJ are separated from species PV, SI, SB, and ZM with high confidence (BS 72–100), where the higher BS corresponds to the *full-metabolites-set* feature vector. On the other hand, the branches of species SB and ZM (BS 79–100) obtained high confidence values when using the *common-metabolites-set* as a feature vector.

Moreover, we also analyzed the robustness of the method according to the number of metabolites composing the feature vector *ϕ*_*P*_ = {*μ*_1_, *μ*_2_, …, *μ*_*m*_}, i.e. in order to analyze the adequacy of the characters (metabolites) within this vector. Therefore, we built various samples containing, for example, 100, 200, …, 4500 characters in order to build different feature vectors, and then, we applied a bootstrap-re-sampling with 100 bootstrap rounds (see Material and methods section). The results of this analysis are found in Fig. 5b, where each curve (color) corresponds to the branches found in Fig. 5a. The curves corresponding to the branches with the highest bootstrap show that this feature vector is able to distinguish between major clades with a small number of characters, while, for some other branches, a bigger number of characters are needed to reach a reasonable support value.

## Discussions

We have analyzed the topological properties from 17 plant metabolic networks modeled from the metabolic reactions found at the PlantCyc database aiming to trace evolutionary information from these species. Initially, the number of reactions and the number of nodes (metabolites) in each plant metabolic network were useful to demonstrate the close relationship between their properties and their evolutionary pattern, as already suggested by Chae *et al*.^14^. Although this previous work showed that the metabolic network sizes have an evident correlation with the evolutionary divergence at plant kingdom, the same is not true when considering the size of plant genomes. For instance, the size of the nuclear genome of *Arabidopsis thaliana* has 125 megabases (Mb) and *Physcomitrella patens* has 480 Mb with 25,498 and 35,938 genes, respectively^23,24^; However, the number of metabolic reactions of these plants is 3424 and 2651, respectively. Consequently, the number of genes per plant is not related to the size of the metabolic network. Preliminary data regarding the metabolic network size raised the question: has the topological structure of a metabolic network been modified in accordance with the evolutionary divergence of plants?

Besides the fact that metabolic networks, in general, have invariable global topological properties such as the scale-free nature^18^, it confirms that the same highly connected network nodes (hubs) may provide the relation between key metabolites responsible for distinct metabolic functions^18^. We have shown in this manuscript, that metabolic network posses intrinsic structural patterns that allow being more grouped between similar classes and more disperse from the unrelated ones. We have demonstrated that, conversely, some local topological network measurements (such as hub-score, degree, in-degree, and out-degree) showed variation within the major plant clades.

The dendrogram presented in Fig. 3 shows that the analysis performed using hub-score seems to reflect plant phylogeny, since the major groups *Chlorophytes* (*Chlamydomonas*), *Bryophytes* (*Physcomitrella*), *Lycophytes* (*Selaginella*), *Dicotyledons*, and *Monocotyledons* are well separated. These results were supported by both the cophenetic correlation and bootstrap analysis, regarding the phylogenetic distance based on the DNA sequence alignment of chloroplast genes^2^, which correctly establish the taxonomy of 17 studied species. The explanations for such separation are rather complex. However, some more general features of metabolism can help explain them from a broader viewpoint. One possible trend that might help to explain the pattern of the grouping of species found in the present work is the gradual evolution of secondary metabolism during the evolution of plants^25^. One first step from *Chlorophytes* to *Bryophytes*/*Lycophytes* could be possibly associated with the evolution of the capacity to make lignin by plants from the latter groups. In the Angiosperm groups (represented here by monocots and dicots), secondary metabolism evolved to produce volatile compounds related to plant communication and defense. Thus, the dendrogram that results from hub-score analysis observed in, for instance, the difference in structure, as well as the number of reactions likely reflect the complexity of metabolism.

The separation of species from Dicots and Monocots is more difficult to explain. The differences might be related to the different genes and corresponding reactions present in both groups of plants^26^. The secondary metabolism of plants of the two groups are different, but equally complex. Some parts of the metabolism (e.g., flavonoids) have even been demonstrated to complement each other^27^. It also is possible that in the case of *Zea*, *Sorghum*, and *Brachypodium*, the presence of the C4 photosynthesis might have made a difference. Wang *et al*.^28^ found that important differences in the topology of the metabolic network of a C3 (*Arabidopsis*) and the C4 grass maize (*Zea*), the latter being considered denser, with higher robustness, and better modularity. Such differences might also have influenced the variations found in the present work.

Regarding the technical aspects of the proposed methodology, it is worth to notice that a reduced portion of the metabolic structure, i.e. 1880 metabolites that are common among all the studied plants (*common-metabolites-set*), was able to establish taxonomic correlations by means of topological measures of metabolic networks. This fact indicates that many other modeling strategies can be used to extract more accurate models, thus, allowing better pattern analysis. Besides that, regarding the point of using all the metabolites or just the common ones, we have observed that it is possible to separate monocots and dicots with 100 Bootstrap rounds by using only the *common-metabolites-set* (*m* = 1880), notwithstanding, when going throughout the subclades this BS falls. However, on the other hand, when considering the *full-metabolites-set* (*m* = 4583), the correct assignment of subclades is improved. This suggests that important evolutionary information is also present in specific metabolites. Moreover, as shown in Fig. 5b, the number of metabolites is related to the performance of the bootstrap analysis, which indicates that the more metabolites considered to compound the feature vector the more confidence compared to the phylogenetic tree DNA can be obtained. Moreover, this also suggests that evolutionary information is present in *common-metabolites-set* as much as in the full content of metabolites of each plant.

One of the main advantages of the proposed method is the possibility to reconstruct phylogenetic trees based on simple topological measures extracted from the metabolic machinery (e.g. a feature vector extracted from the hubs-score), which consequently can be considered as a complementary tool for plant phylogenetic analysis. In this manner, different approaches or visions can provide extra information that can be used to corroborate the study of evolution, that apart from being largely studied is not fully understanding yet. Another advantage is that the metabolic network model studied in this work is considered a minimalist approximation of metabolic networks since they were constructed only by considering general aspects of the biochemical reactions. Due to this fact, we believed that many other scenarios of molecular networks can be also analyzed such as enzymes networks, genes, and proteins networks, since they may have rich information within their topological structure. This fact indicates that many other network modeling strategies, for instances, stoichiometric coefficients, and enzymes catalyzers, which could be explored in future investigations, can be used to extract yet more accurate phylogenetic correlations and pattern analysis.

On the other hand, one of the limitations of this approach is that it relies on the set of metabolic reactions, which consequently depends on the completeness of the content of the accessible databases^29^. Besides, PlantCyc contains a trustful reconstruction of the plant biochemical pathways, as it was used in many works^30–36^, perhaps future metabolic databases with more complete data would carry out an improvement of the results obtained in this work.

Finally, our findings call for more in-depth comparative analyses of the metabolic pathways of plants of these groups to try pointing out to functional features and to the possibility of including the metabolic networks in plant phylogenetic analysis–and also to other types of organisms–in the future. Not only these initial hypotheses related to metabolic networks evolution can be tested and possibly extended, but this could also open interesting opportunities to implement synthetic biology strategies for plants.

## Material and Methods

**Figure 6:**
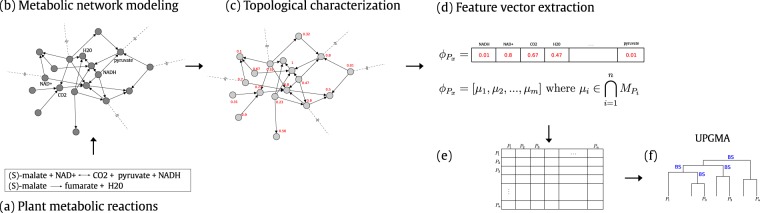
Overview of the proposed method. Each block represents a processing step. (**a**) An example of two metabolic reactions. (**b**) The metabolic network modeling considers the substrate-products network model, where each metabolite from substrates is linked to each metabolite from product reactions. (**c**) Local topological metrics are calculated for each network, thus each metabolite is represented by a value. (**d**) The measurements calculated previously are used to compound one feature vector per each metabolic network. (**e**) The distance matrix contains the Euclidean distances among pairs of plants. (**f**) Construction of a dendrogram using the UPGMA clustering method, which contains evolutionary information about the studied plants.

Figure 6 shows the proposed method to extract evolutionary information from plant metabolic networks. First, some preprocessing of the dataset of metabolic reactions is required in order to build a metabolic network (Fig. 6a,b). Later on, the local topological measures are calculated (Fig. 6c), to later proceed with the feature extraction and dimensionality reduction (Fig. 6d), to finally construct a hierarchical clustering (Fig. 6e) to validate them by comparisons using bootstrap support with an appropriate phylogenetic reconstruction data (“guide tree”).

### Metabolic reactions database

The plant metabolic networks studied here were constructed from the metabolic reactions of 17 plants available at PlantCyc database^19^ (version 9.5). Each plant reaction dataset and its corresponding web references are shown in Table 1. This database contains the reconstruction of the plant biochemical pathways based on enzyme sequence from annotated metabolic functions of proteins sequences^30–35^ (curated dataset available at Supplementary Dataset S1–S3).

**Table 1:** List of web references of the reaction dataset for the 17 plants used in this study corresponding to PlantCyc (version 9.5)^19^ is available.

Plant	Database of reactions
Brachypodium distachyon	https://pmn.plantcyc.org/BRACHYPODIUM/class-instances?object=Reactions
Hordeum vulgare	https://pmn.plantcyc.org/BARLEY/class-instances?object=Reactions
Oryza sativa japonica	https://pmn.plantcyc.org/ORYZA/class-instances?object=Reactions
Panicum virgatum	https://pmn.plantcyc.org/SWITCHGRASS/class-instances?object=Reactions
Setaria italica	https://pmn.plantcyc.org/SETARIA/class-instances?object=Reactions
Sorghum bicolor	https://pmn.plantcyc.org/SORGHUMBICOLOR/class-instances?object=Reactions
Zea mays	https://pmn.plantcyc.org/CORN/class-instances?object=Reactions
Arabidopsis thaliana col	https://pmn.plantcyc.org/ARA/class-instances?object=Reactions
Brassica rapa pekinensis	https://pmn.plantcyc.org/CHINESECABBAGE/class-instances?object=Reactions
Carica papaya	https://pmn.plantcyc.org/PAPAYA/class-instances?object=Reactions
Glycine max	https://pmn.plantcyc.org/SOY/class-instances?object=Reactions
Manihot esculenta	https://pmn.plantcyc.org/CASSAVA/class-instances?object=Reactions
Populus trichocarpa	https://pmn.plantcyc.org/POPLAR/class-instances?object=Reactions
Vitis vinifera	https://pmn.plantcyc.org/GRAPE/class-instances?object=Reactions
Selaginella moellendorffii	https://pmn.plantcyc.org/SELAGINELLA/class-instances?object=Reactions
Physcomitrella patens	https://pmn.plantcyc.org/MOSS/class-instances?object=Reactions
Chlamydomonas reinhardtii	https://pmn.plantcyc.org/CHLAMY/class-instances?object=Reactions

Prior to modeling the data into a network, some preprocessing steps were applied. (i) The database was converted to UTF-8 codification in order to avoid invalid characters; (ii) The duplicated biochemical formulas were removed; (iii) The reactions were standardized, for example, NADP(H) was referred as NADPH and (iv) The stoichiometric coefficient that precedes biochemical formulas were removed, since they were not contemplated in this study. With this latter preprocessing, therefore, we have means to model a metabolic network.

### Metabolic network modeling

The plant metabolic networks were modeled using the set of biochemical reactions from a specific plant. Moreover, we followed the substrate-product network model^37^, where metabolites (nodes) from substrates are linked to metabolites from products of the reactions (dataset available at Supplementary Dataset S1–S3). Figure 6a,b shows an example of the metabolic network modeling. For illustration’s sake, the plot shows two metabolic reactions that correspond to the network modeling on top. This network is represented by a directed graph *G*(*V*, *E*), where *V* is a set of *N* metabolites (nodes) linked by a set of edges *E*. Moreover, each metabolic reaction directionality was considered. Thus, each metabolite is individually linked to each other with respect to the reaction arrows (left ⇒ and right ⇐ and bidirectional ⇔). In the case of the bidirectional arrows, two links are considered separately (incoming and outgoing directions). Noticed that as *V* and *E* are set, therefore repeated metabolites and/or edges are dismissed.The metabolic network is represented by the adjacency matrix *A* of *N* × *N*, where each element *a*_*ij*_ is related to each other by a link.

### Datasets

#### Common-metabolites-set

This dataset contains *m* = 1880 metabolites representing those metabolite with high connectivity such as ADP, ammonia, AMP, ATP, CO _2_, among others. This dataset is composed by the intersection among all metabolites of plants analyzed here (Supplementary Dataset S4).

#### Full-metabolites-set

This dataset is composed by the total of metabolites (*m* = 4583) found in the 17 plants studied (Supplementary Dataset S5).

#### Common-reactions-set

This dataset is composed by the common reactions (1149) among the 17 plants being studied (Supplementary Dataset S6).

### Topological characterization

The topological metrics used to characterize metabolic networks are described in the sequel. The degree of node *v*_*i*_ is represented by *k*_*i*_, also called connectivity, which is the total number of incoming links and outgoing links, where ${k}_{i}^{{\rm{in}}}$ and ${k}_{i}^{{\rm{out}}}$ are the in- and out-degree, respectively given by ${k}_{i}={k}_{i}^{{\rm{in}}}+{k}_{i}^{{\rm{out}}}={\sum }_{j=1}^{N}{a}_{ij}+{\sum }_{j=1}^{N}{a}_{ji}$, and, the average degree 〈$\bar{k}$〉 of a network is given by

1 $$\langle \bar{k}\rangle =\frac{1}{N}\sum _{i=1}^{N}{k}_{i}^{{\rm{in}}}=\frac{1}{N}\sum _{i=1}^{N}{k}_{i}^{{\rm{out}}}=\frac{1}{N}\sum _{ij}^{N}{a}_{ij}\mathrm{.}$$

The local clustering coefficient C_*i*_ of node *v*_*i*_ is the probability that two neighbors of *v*_*i*_ are connected each other forming a triangle^17^, calculated as

2 $${{\rm{C}}}_{i}=\frac{{e}_{i}}{{k}_{i}({k}_{i}-\mathrm{1)}},$$

where *e*_*i*_ is the number of connected pairs between all neighbors of *v*_*i*_. The shortest distance between two metabolites (nodes) is given by *D*(*v*_*i*_, *v*_*j*_), which assumes zero when it is impossible to reach *v*_*j*_ from *v*_*i*_. The average path length *L* of a network is calculated by finding the shortest path between all pairs of metabolites given by

3 $$L=\sum _{i\ne j}\,\frac{D({v}_{i},{v}_{j})}{N(N-\mathrm{1)}}.$$

The in- and out-degree distribution *P*(*k*) is assumed as the probability of having a node with *k*^in^ and *k*^out^ degrees respectively^17^. Scale-free networks follow a power law distribution, $P(k) \sim {k}^{-\gamma }$ where *γ*_in_ or *γ*_out_ are the incoming and outgoing power-law exponent respectively.

According to the Kleinberg’s HITS algorithm^38^, there are two types of nodes: hubs and authorities. Hubs are nodes highly connected, on the other hand authorities are nodes linked by many hubs. HITS algorithm assigns scores to hubs H(*v*_*i*_) and authorities A(*v*_*i*_), and computed them based on the node degree *k*_*i*_ considering the participation of each node and their mutually reinforcement. This algorithm assumes the initial condition H(*v*_*i*_) = A(*v*_*i*_) = 1, as established by the equations

4 $${\rm{A}}({v}_{j})=\sum _{{a}_{ij=1}}\,{\rm{H}}({v}_{i}),\,{\rm{and}}\,{\rm{H}}({v}_{i})=\sum _{{a}_{ij=1}}\,{\rm{A}}({v}_{j}\mathrm{).}$$

We also considered two centrality measures, i.e. the betweenness and the eigenvector. The former metric considers the importance of a node regarding the information load it is responsible in the network^39^. The communication between two nodes *j* and *k*, that are not adjacent, depends on the nodes that belong to the paths that connect *j* and *k*, i.e. acting as a bridge. Thus, the betweenness B_*i*_ of a node *i* is computed as a function of the number of shortest paths passing through *i*^39^:

5 $${{\rm{B}}}_{i}=\frac{1}{(N-\mathrm{1)(}N-\mathrm{2)}}\,\sum _{j,k,j\ne k}\,\frac{{\sigma }_{jk}(i)}{{\sigma }_{jk}},$$

where *σ*_*jk*_(*i*) is the number of geodesic paths connecting *j* and *k* passing through *i* and *σ*_*jk*_ is the number of geodesic paths connecting *j* and *k*.

Regarding the eigenvector centrality, or simply eigencentrality E_*i*_^40^, it is a metric that scores the influence of a node in a network. Thus, for instance, highest values represent those nodes which are connected to many other nodes which are, similarly, connected to many others. The eigencentrality metric is calculated using the highest eigenvalue *λ* given by the adjacency matrix *A* and corresponding eigenvector *X*^40^, as follows:

6 $${{\rm{E}}}_{{\rm{i}}}=\frac{1}{\lambda }A\cdot X$$

Another metric used for network analysis is the efficiency, which can be applied in both local and global scales. The global efficiency *ε* measures how efficiently information is exchanged over the network^41^, while the local efficiency F_*i*_ accounts for the network’s tolerance when node *i* is removed, characterizing how well information is exchanged by its neighborhood, and it is defined as:

7 $${{\rm{F}}}_{i}=\frac{1}{N}\,\sum _{i}^{N}\,\varepsilon ({v}_{i})$$

where $$\varepsilon ({v}_{i})=\frac{1}{N(N-\mathrm{1)}}\,{\sum }_{i\ne j}\,\frac{1}{D({v}_{i},{v}_{j})}$$.

Should be noticed that all of these metrics can be extracted by means of the network properties, being either local or global, thus, degree, in-degree, out-degree, hub-score, authority-score, local clustering coefficient, local efficiency, betweenness and eigencentrality are local topological measurements, on the other hand, power-law exponent, mean degree and average shortest path are global topological measures.

### Feature extraction

The metabolic networks were characterized in terms of their topological measurements. We adopted local topological measures extracted from each node. Therefore, for each plant metabolic network *M*_*P*_, a feature vector *ϕ*_*P*_ = [*μ*_1_, *μ*_2_, …, *μ*_*m*_] is composed by the concatenation of the calculated measures of each metabolite, where *m* is the number of characters (metabolites). In order to obtain an accurate characterization of the metabolic networks, it was necessary a statistical method for feature normalization or dimensionality reduction, therefore, the principal component analysis (PCA) was employed in order to correlate the plant measurements by using a dimensional projection of the original feature space.

### Dendrograms based on hierarchical clustering

In this work, we assumed that a pair of plants are similar when there exist correlated patterns among their metabolic networks that are also similar to their topological measurements, or vice versa. Thus, after determining the projections of the feature vectors, it was calculated a 17 × 17 distance matrix composed of the Euclidean distances between each pair of feature vector (i.e., a pair of plants), from which a dendrogram can be constructed. In this context, the hierarchical clustering aims to form groups of plants belonging to the same cluster based on their topological characteristics, *i.e*., plants from the same cluster are as similar as possible to each other, while plants from different clusters are dissimilar as possible^42^.

Thus, the UPGMA (Unweighted Pair Group Method with Arithmetic mean) hierarchical clustering method^21^ performed on Matlab 2012a package was employed. In addition, the cophenetic correlation coefficient (CPCC)^42^ is applied in order to evaluate the consistency between two the distance matrices. For our purposes, the distance matrices corresponding to the topological measurements using the *common-metabolites-set* (Supplementary Information S2–S10) and the *full-metabolites-set* (Supplementary Information S11–S20) are contrasted to the distance matrix obtained by the phylogenetic distance matrix based on gene alignment (Supplementary Information S21). The CPCC is a value in the range of [−1, 1], where a value close to 1 indicates a higher Pearson correlation between two clustering structures.

### Bootstrapping analysis of the topological network characters

The bootstrapping method provided by the RAxML software (Randomized Axelerated Maximum Likelihood, version 8.2.11)^22^ was used in order to estimate confidence intervals (BS) in all experiments.

In order to show the adequacy of the number of characters *m* within the feature vector, we constructed ensembles of feature vectors, sampling *m* characters within the 4583 metabolites present in the *full-metabolites-set*. Each ensemble contains 100 random combinations containing different sizes *m* = {100, 200, …, 4500} characters. Therefore, 100 bootstrap replicates were considered per each topological measurement.

### Phylogenetic reconstruction based on 78 plastid genes

In order to generate a phylogenetic reconstruction as a comparison basis for the method proposed here, we have used a recent phylogenetic reconstruction of all plants as a starting point^2^. The matrix containing 78 aligned plastid genes from 360 taxa was pruned to generate an alignment with 15 taxa that included the plants used in this study that are present in the PlantCyc database. For the remaining two taxa (*Brassica rapa pekinensis* and *Setaria italica*), the complete plastid genome was downloaded and the 78 relevant genes were aligned with the previous 15 taxa, using the MAFFT algorithm^43^. This multigene alignment was then subjected to Maximum Likelihood reconstruction using IQTREE^44^. We reconstructed the phylogenetic relationships for all 17 taxa. We first performed the implemented model tool which yielded the following most appropriate models via AIC: JTTDCMut + F + G4 for 17 taxa. We then performed Maximum Likelihood searches using default parameters, followed by 1000 rapid bootstraps to determine support. Resulting trees (Supplementary Dataset S7) with bootstrap supports are concordant with each other and identical to the tree generated by Rufehl and colleagues^2^ if other taxa than those used in this study were removed.

## Electronic supplementary material

Supplementary Information

Supplementary Dataset
