# Supplementary material for: Topological assessment of metabolic networks reveals evolutionary information
Source: Sci Rep. 2018 Oct 29;8:15918. doi: 10.1038/s41598-018-34163-7 (PMC6206017; doi:10.1038/s41598-018-34163-7)

Supplementary Tables

Topological assessment of metabolic networks reveals evolutionary information

Jeaneth Machicao, Humberto A. Filho, Daniel J. G. Lahr, Marcos Buckeridge, and Odemir M. Bruno

Table S1: Metrics of the 17 plant metabolic networks (left side) and their respective topological network metrics (right side) namely the average degree <k>, the shortest path length L, the incoming and outgoing power-law exponent gamma_in, gamma_out, and the respective averages according to their plant clades.

Clade	Plant Specie	Plant Specie	Reactions	Nodes	Edges	$< k >$	L	γ_{in}	γ_{out}
Monocotyledon	*BrachypodiumDistachyon*	BD	2855	3107	14446	4,65	3,17	2,86	2,78
	HordeumVulgare	HV	2837	3059	14468	4,73	3,13	2,85	2,78
	OryzaSativaJaponica	OSJ	2936	3178	14786	4,65	3,18	2,86	2,78
	PanicumVirgatum	PV	2920	3165	14840	4,69	3,15	2,86	2,78
	SetariaItalica	SI	2878	3111	14606	4,69	3,13	2,84	2,76
	SorghumBicolor	SB	2877	3108	14480	4,66	3,15	2,85	2,78
	ZeaMays	ZM	2898	3146	14535	4,62	3,14	2,88	2,8
Dicotyledon	*ArabidopsisThalianaCol*	AT	3424	3546	16371	4,62	3,18	2,82	2,73
	BrassicaRapaPekinensis	BRP	3041	3249	15030	4,62	3,17	2,83	2,77
	CaricaPapaya	CP	2935	3155	14655	4,64	3,16	2,86	2,78
	GlycineMax	GM	3041	3242	15219	4,69	3,15	2,85	2,77
	ManihotEsculenta	ME	2991	3210	15100	4,71	3,16	2,84	2,76
	PopulusTrichocarpa	PT	3056	3274	15452	4,72	3,14	2,84	2,75
	VitisVinifera	VV	2954	3197	15012	4,70	3,16	2,76	2,76
Lycophytes	*SelaginellaMoellendorffii*	SM	2675	2924	13866	4,74	3,15	2,81	2,77
Bryophytes	*PhyscomitrellaPatens*	PP	2651	2858	13730	4,80	3,13	2,79	2,74
Chlorophytes	*ChlamydomonasReinhardtii*	CR	2208	2433	11516	4,73	3,15	2,79	2,8

Table S2: Distance matrix derived from the **hub-score** measure using the *commom-metabolites-set* **.** Each pair represent the distance formed among each pair of the 17 plants species.

		BD	HV	OSJ	PV	SI	SB	ZM	AT	BRP	CP	GM	ME	PT	VV	SM	PP	CR
BrachypodiumDistachyon	BD	0,00000	0,00900	0,00800	0,01000	0,01600	0,00300	0,00400	0,02600	0,01600	0,01600	0,02900	0,02400	0,02500	0,01800	0,06000	0,09100	0,25500
HordeumVulgare	HV	0,00900	0,00000	0,00300	0,00300	0,00700	0,00900	0,00800	0,02100	0,01200	0,01300	0,02000	0,01600	0,01700	0,01100	0,06900	0,09900	0,26400
OryzaSativaJaponica	OSJ	0,00800	0,00300	0,00000	0,00300	0,01000	0,00700	0,00500	0,01900	0,01000	0,01000	0,02100	0,01700	0,01700	0,01100	0,06700	0,09700	0,26200
PanicumVirgatum	PV	0,01000	0,00300	0,00300	0,00000	0,00700	0,01000	0,00800	0,01800	0,01000	0,01100	0,01900	0,01400	0,01500	0,00900	0,07000	0,10000	0,26400
SetariaItalica	SI	0,01600	0,00700	0,01000	0,00700	0,00000	0,01600	0,01500	0,02000	0,01500	0,01600	0,01300	0,01100	0,01400	0,00900	0,07600	0,10700	0,27100
SorghumBicolor	SB	0,00300	0,00900	0,00700	0,01000	0,01600	0,00000	0,00200	0,02400	0,01400	0,01300	0,02800	0,02400	0,02300	0,01700	0,06000	0,09000	0,25500
ZeaMays	ZM	0,00400	0,00800	0,00500	0,00800	0,01500	0,00200	0,00000	0,02200	0,01200	0,01200	0,02700	0,02200	0,02100	0,01500	0,06200	0,09200	0,25700
ArabidopsisThalianaCol	AT	0,02600	0,02100	0,01900	0,01800	0,02000	0,02400	0,02200	0,00000	0,01000	0,01100	0,01900	0,01400	0,00800	0,01100	0,07800	0,10700	0,27100
BrassicaRapaPekinensis	BRP	0,01600	0,01200	0,01000	0,01000	0,01500	0,01400	0,01200	0,01000	0,00000	0,00200	0,02100	0,01500	0,01200	0,00800	0,07000	0,10000	0,26400
CaricaPapaya	CP	0,01600	0,01300	0,01000	0,01100	0,01600	0,01300	0,01200	0,01100	0,00200	0,00000	0,02300	0,01700	0,01300	0,01000	0,06800	0,09800	0,26200
GlycineMax	GM	0,02900	0,02000	0,02100	0,01900	0,01300	0,02800	0,02700	0,01900	0,02100	0,02300	0,00000	0,00600	0,01100	0,01300	0,08800	0,11900	0,28300
ManihotEsculenta	ME	0,02400	0,01600	0,01700	0,01400	0,01100	0,02400	0,02200	0,01400	0,01500	0,01700	0,00600	0,00000	0,00600	0,00700	0,08300	0,11300	0,27800
PopulusTrichocarpa	PT	0,02500	0,01700	0,01700	0,01500	0,01400	0,02300	0,02100	0,00800	0,01200	0,01300	0,01100	0,00600	0,00000	0,00600	0,08100	0,11100	0,27600
VitisVinifera	VV	0,01800	0,01100	0,01100	0,00900	0,00900	0,01700	0,01500	0,01100	0,00800	0,01000	0,01300	0,00700	0,00600	0,00000	0,07600	0,10600	0,27100
SelaginellaMoellendorffii	SM	0,06000	0,06900	0,06700	0,07000	0,07600	0,06000	0,06200	0,07800	0,07000	0,06800	0,08800	0,08300	0,08100	0,07600	0,00000	0,03000	0,19500
PhyscomitrellaPatens	PP	0,09100	0,09900	0,09700	0,10000	0,10700	0,09000	0,09200	0,10700	0,10000	0,09800	0,11900	0,11300	0,11100	0,10600	0,03000	0,00000	0,16500
ChlamydomonasReinhardtii	CR	0,25500	0,26400	0,26200	0,26400	0,27100	0,25500	0,25700	0,27100	0,26400	0,26200	0,28300	0,27800	0,27600	0,27100	0,19500	0,16500	0,00000

Table S3: Distance matrix derived from the local **clustering coefficient** measure using the *commom-metabolites-set* . Each pair represent the distance formed among each pair of the 17 plants species.

		BD	HV	OSJ	PV	SI	SB	ZM	AT	BRP	CP	GM	ME	PT	VV	SM	PP	CR
BrachypodiumDistachyon	BD	0,00000	8,25113	6,81333	3,54830	4,10589	7,01649	3,40613	3,18232	4,26437	4,62118	5,91106	4,27582	0,53883	3,85955	6,06122	3,80708	3,47236
HordeumVulgare	HV	8,25113	0,00000	1,43920	4,72050	4,16209	1,25096	4,87375	5,15500	3,98719	3,63575	2,34975	4,00886	7,71329	4,39179	2,21072	4,46914	4,93197
OryzaSativaJaponica	OSJ	6,81333	1,43920	0,00000	3,29098	2,73465	0,31566	3,43544	3,72107	2,54897	2,19656	0,91304	2,57279	6,27524	2,95493	0,78378	3,03100	3,50734
PanicumVirgatum	PV	3,54830	4,72050	3,29098	0,00000	0,55921	3,47678	0,62469	0,94157	0,78925	1,15960	2,41202	0,96413	3,01518	0,39988	2,57197	0,64140	1,06504
SetariaItalica	SI	4,10589	4,16209	2,73465	0,55921	0,00000	2,91767	0,93017	1,28486	0,34857	0,66784	1,86457	0,65639	3,57175	0,33411	2,02693	0,65902	1,27038
SorghumBicolor	SB	7,01649	1,25096	0,31566	3,47678	2,91767	0,00000	3,66265	3,95994	2,75931	2,41933	1,16473	2,81038	6,47978	3,15847	1,05664	3,26011	3,75960
ZeaMays	ZM	3,40613	4,87375	3,43544	0,62469	0,93017	3,66265	0,00000	0,35492	0,92398	1,24335	2,52412	0,87102	2,86784	0,59744	2,66767	0,40475	0,44696
ArabidopsisThalianaCol	AT	3,18232	5,15500	3,72107	0,94157	1,28486	3,95994	0,35492	0,00000	1,25637	1,54964	2,80805	1,14969	2,64852	0,95160	2,94432	0,71350	0,32623
BrassicaRapaPekinensis	BRP	4,26437	3,98719	2,54897	0,78925	0,34857	2,75931	0,92398	1,25637	0,00000	0,37157	1,64993	0,32997	3,72627	0,41100	1,80461	0,54695	1,15375
CaricaPapaya	CP	4,62118	3,63575	2,19656	1,15960	0,66784	2,41933	1,24335	1,54964	0,37157	0,00000	1,28995	0,42030	4,08255	0,78093	1,44091	0,84187	1,38876
GlycineMax	GM	5,91106	2,34975	0,91304	2,41202	1,86457	1,16473	2,52412	2,80805	1,64993	1,28995	0,00000	1,65985	5,37246	2,05956	0,16720	2,11945	2,59772
ManihotEsculenta	ME	4,27582	4,00886	2,57279	0,96413	0,65639	2,81038	0,87102	1,14969	0,32997	0,42030	1,65985	0,00000	3,73724	0,56686	1,79962	0,46887	0,97011
PopulusTrichocarpa	PT	0,53883	7,71329	6,27524	3,01518	3,57175	6,47978	2,86784	2,64852	3,72627	4,08255	5,37246	3,73724	0,00000	3,32210	5,52246	3,26846	2,94267
VitisVinifera	VV	3,85955	4,39179	2,95493	0,39988	0,33411	3,15847	0,59744	0,95160	0,41100	0,78093	2,05956	0,56686	3,32210	0,00000	2,21511	0,35640	0,94480
SelaginellaMoellendorffii	SM	6,06122	2,21072	0,78378	2,57197	2,02693	1,05664	2,66767	2,94432	1,80461	1,44091	0,16720	1,79962	5,52246	2,21511	0,00000	2,26298	2,72487
PhyscomitrellaPatens	PP	3,80708	4,46914	3,03100	0,64140	0,65902	3,26011	0,40475	0,71350	0,54695	0,84187	2,11945	0,46887	3,26846	0,35640	2,26298	0,00000	0,62093
ChlamydomonasReinhardtii	CR	3,47236	4,93197	3,50734	1,06504	1,27038	3,75960	0,44696	0,32623	1,15375	1,38876	2,59772	0,97011	2,94267	0,94480	2,72487	0,62093	0,00000

Table S4: Distance matrix derived from the **degree** measure using the *commom-metabolites-set*. Each pair represent the distance formed among each pair of the 17 plants species.

		BD	HV	OSJ	PV	SI	SB	ZM	AT	BRP	CP	GM	ME	PT	VV	SM	PP	CR
BrachypodiumDistachyon	BD	0,00000	0,01724	0,02598	0,04056	0,02487	0,01076	0,01054	0,13810	0,05118	0,03295	0,07561	0,07471	0,10265	0,06524	0,07014	0,09346	0,32720
HordeumVulgare	HV	0,01724	0,00000	0,00880	0,02332	0,00832	0,00992	0,00846	0,12110	0,03394	0,01570	0,05891	0,05748	0,08548	0,04811	0,08655	0,10873	0,34437
OryzaSativaJaponica	OSJ	0,02598	0,00880	0,00000	0,01474	0,00300	0,01737	0,01638	0,11282	0,02534	0,00716	0,05108	0,04885	0,07700	0,03976	0,09528	0,11719	0,35316
PanicumVirgatum	PV	0,04056	0,02332	0,01474	0,00000	0,01665	0,03205	0,03112	0,09813	0,01063	0,00762	0,03687	0,03416	0,06226	0,02507	0,10901	0,12995	0,36754
SetariaItalica	SI	0,02487	0,00832	0,00300	0,01665	0,00000	0,01551	0,01477	0,11476	0,02710	0,00937	0,05335	0,05047	0,07877	0,04172	0,09469	0,11704	0,35202
SorghumBicolor	SB	0,01076	0,00992	0,01737	0,03205	0,01551	0,00000	0,00185	0,13018	0,04258	0,02453	0,06838	0,06598	0,09426	0,05711	0,08070	0,10422	0,33687
ZeaMays	ZM	0,01054	0,00846	0,01638	0,03112	0,01477	0,00185	0,00000	0,12919	0,04170	0,02354	0,06722	0,06517	0,09337	0,05613	0,08068	0,10391	0,33732
ArabidopsisThalianaCol	AT	0,13810	0,12110	0,11282	0,09813	0,11476	0,13018	0,12919	0,00000	0,08769	0,10566	0,06281	0,06489	0,03653	0,07307	0,20229	0,21870	0,46216
BrassicaRapaPekinensis	BRP	0,05118	0,03394	0,02534	0,01063	0,02710	0,04258	0,04170	0,08769	0,00000	0,01825	0,02742	0,02354	0,05168	0,01480	0,11931	0,13977	0,37808
CaricaPapaya	CP	0,03295	0,01570	0,00716	0,00762	0,00937	0,02453	0,02354	0,10566	0,01825	0,00000	0,04401	0,04178	0,06986	0,03260	0,10169	0,12303	0,36000
GlycineMax	GM	0,07561	0,05891	0,05108	0,03687	0,05335	0,06838	0,06722	0,06281	0,02742	0,04401	0,00000	0,01498	0,02960	0,01339	0,13988	0,15761	0,39974
ManihotEsculenta	ME	0,07471	0,05748	0,04885	0,03416	0,05047	0,06598	0,06517	0,06489	0,02354	0,04178	0,01498	0,00000	0,02847	0,01095	0,14238	0,16203	0,40150
PopulusTrichocarpa	PT	0,10265	0,08548	0,07700	0,06226	0,07877	0,09426	0,09337	0,03653	0,05168	0,06986	0,02960	0,02847	0,00000	0,03744	0,16890	0,18713	0,42859
VitisVinifera	VV	0,06524	0,04811	0,03976	0,02507	0,04172	0,05711	0,05613	0,07307	0,01480	0,03260	0,01339	0,01095	0,03744	0,00000	0,13198	0,15127	0,39140
SelaginellaMoellendorffii	SM	0,07014	0,08655	0,09528	0,10901	0,09469	0,08070	0,08068	0,20229	0,11931	0,10169	0,13988	0,14238	0,16890	0,13198	0,00000	0,02729	0,25989
PhyscomitrellaPatens	PP	0,09346	0,10873	0,11719	0,12995	0,11704	0,10422	0,10391	0,21870	0,13977	0,12303	0,15761	0,16203	0,18713	0,15127	0,02729	0,00000	0,24527
ChlamydomonasReinhardtii	CR	0,32720	0,34437	0,35316	0,36754	0,35202	0,33687	0,33732	0,46216	0,37808	0,36000	0,39974	0,40150	0,42859	0,39140	0,25989	0,24527	0,00000

Table S5: Distance matrix derived from the **in-degree** measure using the *commom-metabolites-set*. Each pair represent the distance formed among each pair of the 17 plants species.

		BD	HV	OSJ	PV	SI	SB	ZM	AT	BRP	CP	GM	ME	PT	VV	SM	PP	CR
BrachypodiumDistachyon	BD	0,00000	0,01570	0,02597	0,03724	0,02000	0,01135	0,00988	0,14190	0,05843	0,04055	0,07373	0,07501	0,10098	0,06542	0,06446	0,08645	0,30814
HordeumVulgare	HV	0,01570	0,00000	0,01031	0,02163	0,00692	0,00788	0,00618	0,12622	0,04329	0,02495	0,05806	0,05977	0,08540	0,04993	0,07783	0,09838	0,32299
OryzaSativaJaponica	OSJ	0,02597	0,01031	0,00000	0,01133	0,00844	0,01652	0,01621	0,11593	0,03324	0,01465	0,04776	0,04962	0,07509	0,03966	0,08748	0,10735	0,33309
PanicumVirgatum	PV	0,03724	0,02163	0,01133	0,00000	0,01829	0,02723	0,02741	0,10469	0,02226	0,00332	0,03652	0,03842	0,06377	0,02834	0,09842	0,11774	0,34431
SetariaItalica	SI	0,02000	0,00692	0,00844	0,01829	0,00000	0,00911	0,01031	0,12266	0,03852	0,02146	0,05459	0,05512	0,08143	0,04579	0,08375	0,10476	0,32813
SorghumBicolor	SB	0,01135	0,00788	0,01652	0,02723	0,00911	0,00000	0,00377	0,13175	0,04757	0,03046	0,06366	0,06418	0,09054	0,05489	0,07574	0,09744	0,31935
ZeaMays	ZM	0,00988	0,00618	0,01621	0,02741	0,01031	0,00377	0,00000	0,13210	0,04857	0,03071	0,06393	0,06515	0,09111	0,05554	0,07350	0,09478	0,31788
ArabidopsisThalianaCol	AT	0,14190	0,12622	0,11593	0,10469	0,12266	0,13175	0,13210	0,00000	0,08506	0,10139	0,06817	0,06904	0,04167	0,07704	0,19944	0,21483	0,44605
BrassicaRapaPekinensis	BRP	0,05843	0,04329	0,03324	0,02226	0,03852	0,04757	0,04857	0,08506	0,00000	0,01913	0,01911	0,01661	0,04344	0,00869	0,12065	0,13992	0,36628
CaricaPapaya	CP	0,04055	0,02495	0,01465	0,00332	0,02146	0,03046	0,03071	0,10139	0,01913	0,00000	0,03323	0,03516	0,06045	0,02503	0,10164	0,12080	0,34759
GlycineMax	GM	0,07373	0,05806	0,04776	0,03652	0,05459	0,06366	0,06393	0,06817	0,01911	0,03323	0,00000	0,01202	0,02774	0,01045	0,13290	0,15028	0,37951
ManihotEsculenta	ME	0,07501	0,05977	0,04962	0,03842	0,05512	0,06418	0,06515	0,06904	0,01661	0,03516	0,01202	0,00000	0,02739	0,01077	0,13676	0,15546	0,38271
PopulusTrichocarpa	PT	0,10098	0,08540	0,07509	0,06377	0,08143	0,09054	0,09111	0,04167	0,04344	0,06045	0,02774	0,02739	0,00000	0,03565	0,16062	0,17772	0,40726
VitisVinifera	VV	0,06542	0,04993	0,03966	0,02834	0,04579	0,05489	0,05554	0,07704	0,00869	0,02503	0,01045	0,01077	0,03565	0,00000	0,12637	0,14481	0,37257
SelaginellaMoellendorffii	SM	0,06446	0,07783	0,08748	0,09842	0,08375	0,07574	0,07350	0,19944	0,12065	0,10164	0,13290	0,13676	0,16062	0,12637	0,00000	0,02476	0,24672
PhyscomitrellaPatens	PP	0,08645	0,09838	0,10735	0,11774	0,10476	0,09744	0,09478	0,21483	0,13992	0,12080	0,15028	0,15546	0,17772	0,14481	0,02476	0,00000	0,23224
ChlamydomonasReinhardtii	CR	0,30814	0,32299	0,33309	0,34431	0,32813	0,31935	0,31788	0,44605	0,36628	0,34759	0,37951	0,38271	0,40726	0,37257	0,24672	0,23224	0,00000

Table S6: Distance matrix derived from the **out-degree** measure using the *commom-metabolites-set* **.** Each pair represent the distance formed among each pair of the 17 plants species.

		BD	HV	OSJ	PV	SI	SB	ZM	AT	BRP	CP	GM	ME	PT	VV	SM	PP	CR
BrachypodiumDistachyon	BD	0,00000	0,01652	0,03099	0,04319	0,02901	0,00889	0,02329	0,14384	0,04762	0,02834	0,08414	0,07639	0,10688	0,06595	0,09721	0,12215	0,37842
HordeumVulgare	HV	0,01652	0,00000	0,01813	0,02707	0,01401	0,01322	0,02000	0,12740	0,03144	0,01423	0,06764	0,06011	0,09049	0,04944	0,11109	0,13487	0,39415
OryzaSativaJaponica	OSJ	0,03099	0,01813	0,00000	0,01674	0,00622	0,02323	0,01564	0,11588	0,02069	0,00442	0,05765	0,04825	0,07879	0,04027	0,12800	0,15239	0,40899
PanicumVirgatum	PV	0,04319	0,02707	0,01674	0,00000	0,01478	0,03718	0,03233	0,10086	0,00444	0,01634	0,04165	0,03322	0,06380	0,02381	0,13747	0,16038	0,42122
SetariaItalica	SI	0,02901	0,01401	0,00622	0,01478	0,00000	0,02246	0,01894	0,11555	0,01918	0,00232	0,05642	0,04784	0,07846	0,03850	0,12503	0,14888	0,40742
SorghumBicolor	SB	0,00889	0,01322	0,02323	0,03718	0,02246	0,00000	0,01445	0,13801	0,04161	0,02131	0,07875	0,07030	0,10092	0,06068	0,10573	0,13089	0,38576
ZeaMays	ZM	0,02329	0,02000	0,01564	0,03233	0,01894	0,01445	0,00000	0,13083	0,03633	0,01675	0,07316	0,06347	0,09382	0,05590	0,11915	0,14476	0,39675
ArabidopsisThalianaCol	AT	0,14384	0,12740	0,11588	0,10086	0,11555	0,13801	0,13083	0,00000	0,09642	0,11684	0,05990	0,06774	0,03711	0,07804	0,23305	0,25245	0,51962
BrassicaRapaPekinensis	BRP	0,04762	0,03144	0,02069	0,00444	0,01918	0,04161	0,03633	0,09642	0,00000	0,02065	0,03729	0,02879	0,05936	0,01960	0,14158	0,16430	0,42556
CaricaPapaya	CP	0,02834	0,01423	0,00442	0,01634	0,00232	0,02131	0,01675	0,11684	0,02065	0,00000	0,05794	0,04910	0,07973	0,04014	0,12487	0,14896	0,40673
GlycineMax	GM	0,08414	0,06764	0,05765	0,04165	0,05642	0,07875	0,07316	0,05990	0,03729	0,05794	0,00000	0,01136	0,02371	0,01820	0,17404	0,19465	0,46007
ManihotEsculenta	ME	0,07639	0,06011	0,04825	0,03322	0,04784	0,07030	0,06347	0,06774	0,02879	0,04910	0,01136	0,00000	0,03063	0,01297	0,16895	0,19060	0,45394
PopulusTrichocarpa	PT	0,10688	0,09049	0,07879	0,06380	0,07846	0,10092	0,09382	0,03711	0,05936	0,07973	0,02371	0,03063	0,00000	0,04142	0,19775	0,21828	0,48365
VitisVinifera	VV	0,06595	0,04944	0,04027	0,02381	0,03850	0,06068	0,05590	0,07804	0,01960	0,04014	0,01820	0,01297	0,04142	0,00000	0,15669	0,17797	0,44226
SelaginellaMoellendorffii	SM	0,09721	0,11109	0,12800	0,13747	0,12503	0,10573	0,11915	0,23305	0,14158	0,12487	0,17404	0,16895	0,19775	0,15669	0,00000	0,02796	0,28705
PhyscomitrellaPatens	PP	0,12215	0,13487	0,15239	0,16038	0,14888	0,13089	0,14476	0,25245	0,16430	0,14896	0,19465	0,19060	0,21828	0,17797	0,02796	0,00000	0,27134
ChlamydomonasReinhardtii	CR	0,37842	0,39415	0,40899	0,42122	0,40742	0,38576	0,39675	0,51962	0,42556	0,40673	0,46007	0,45394	0,48365	0,44226	0,28705	0,27134	0,00000

Table S7: Distance matrix derived from the **authority-score** measure using the *commom-metabolites-set*. Each pair represent the distance formed among each pair of the 17 plants species.

		BD	HV	OSJ	PV	SI	SB	ZM	AT	BRP	CP	GM	ME	PT	VV	SM	PP	CR
BrachypodiumDistachyon	BD	0,00000	0,01339	0,01105	0,01362	0,01977	0,00474	0,01419	0,02337	0,01441	0,01234	0,03005	0,03148	0,03434	0,02247	0,06505	0,11474	0,35606
HordeumVulgare	HV	0,01339	0,00000	0,00315	0,00699	0,00867	0,00939	0,02002	0,01519	0,00401	0,00825	0,01865	0,01814	0,02110	0,01018	0,07820	0,12746	0,36845
OryzaSativaJaponica	OSJ	0,01105	0,00315	0,00000	0,00876	0,01172	0,00665	0,01985	0,01791	0,00660	0,00563	0,02180	0,02054	0,02332	0,01333	0,07609	0,12566	0,36684
PanicumVirgatum	PV	0,01362	0,00699	0,00876	0,00000	0,00669	0,01170	0,01456	0,01005	0,00348	0,01438	0,01656	0,02150	0,02476	0,00991	0,07573	0,12409	0,36449
SetariaItalica	SI	0,01977	0,00867	0,01172	0,00669	0,00000	0,01692	0,02054	0,00730	0,00554	0,01684	0,01038	0,01575	0,01907	0,00336	0,08237	0,13053	0,37067
SorghumBicolor	SB	0,00474	0,00939	0,00665	0,01170	0,01692	0,00000	0,01704	0,02174	0,01138	0,00772	0,02730	0,02718	0,02994	0,01920	0,06965	0,11944	0,36079
ZeaMays	ZM	0,01419	0,02002	0,01985	0,01456	0,02054	0,01704	0,00000	0,01924	0,01780	0,02407	0,02826	0,03601	0,03929	0,02390	0,06287	0,11017	0,35013
ArabidopsisThalianaCol	AT	0,02337	0,01519	0,01791	0,01005	0,00730	0,02174	0,01924	0,00000	0,01131	0,02341	0,00918	0,02081	0,02401	0,00897	0,08211	0,12901	0,36816
BrassicaRapaPekinensis	BRP	0,01441	0,00401	0,00660	0,00348	0,00554	0,01138	0,01780	0,01131	0,00000	0,01211	0,01592	0,01857	0,02176	0,00806	0,07805	0,12679	0,36741
CaricaPapaya	CP	0,01234	0,00825	0,00563	0,01438	0,01684	0,00772	0,02407	0,02341	0,01211	0,00000	0,02643	0,02226	0,02454	0,01785	0,07640	0,12653	0,36802
GlycineMax	GM	0,03005	0,01865	0,02180	0,01656	0,01038	0,02730	0,02826	0,00918	0,01592	0,02643	0,00000	0,01481	0,01751	0,00858	0,09107	0,13819	0,37728
ManihotEsculenta	ME	0,03148	0,01814	0,02054	0,02150	0,01575	0,02718	0,03601	0,02081	0,01857	0,02226	0,01481	0,00000	0,00331	0,01256	0,09631	0,14532	0,38596
PopulusTrichocarpa	PT	0,03434	0,02110	0,02332	0,02476	0,01907	0,02994	0,03929	0,02401	0,02176	0,02454	0,01751	0,00331	0,00000	0,01587	0,09929	0,14845	0,38917
VitisVinifera	VV	0,02247	0,01018	0,01333	0,00991	0,00336	0,01920	0,02390	0,00897	0,00806	0,01785	0,00858	0,01256	0,01587	0,00000	0,08564	0,13388	0,37403
SelaginellaMoellendorffii	SM	0,06505	0,07820	0,07609	0,07573	0,08237	0,06965	0,06287	0,08211	0,07805	0,07640	0,09107	0,09631	0,09929	0,08564	0,00000	0,05081	0,29237
PhyscomitrellaPatens	PP	0,11474	0,12746	0,12566	0,12409	0,13053	0,11944	0,11017	0,12901	0,12679	0,12653	0,13819	0,14532	0,14845	0,13388	0,05081	0,00000	0,24165
ChlamydomonasReinhardtii	CR	0,35606	0,36845	0,36684	0,36449	0,37067	0,36079	0,35013	0,36816	0,36741	0,36802	0,37728	0,38596	0,38917	0,37403	0,29237	0,24165	0,00000

Table S8: Distance matrix derived from the **local efficiency** measure using the *commom-metabolites-set* **.** Each pair represent the distance formed among each pair of the 17 plants species.

		BD	HV	OSJ	PV	SI	SB	ZM	AT	BRP	CP	GM	ME	PT	VV	SM	PP	CR
BrachypodiumDistachyon	BD	0,00000	11,64067	10,19937	6,68951	6,82673	10,49465	4,63983	2,55431	3,86560	5,65280	7,94947	5,92075	1,00890	5,34579	8,22858	5,30131	4,74334
HordeumVulgare	HV	11,64067	0,00000	1,44131	5,00607	4,85077	1,19527	7,05654	9,09760	7,77550	6,00313	3,70820	5,73547	12,51143	6,29827	3,41453	6,34422	7,01173
OryzaSativaJaponica	OSJ	10,19937	1,44131	0,00000	3,57686	3,41807	0,43111	5,62126	7,65686	6,33426	4,56440	2,27378	4,29708	11,07140	4,85732	1,97469	4,90369	5,58287
PanicumVirgatum	PV	6,68951	5,00607	3,57686	0,00000	0,17840	3,83105	2,34349	4,19212	2,86577	1,33369	1,30564	1,14372	7,52569	1,38715	1,69893	1,54885	2,41251
SetariaItalica	SI	6,82673	4,85077	3,41807	0,17840	0,00000	3,68071	2,42731	4,31794	2,98890	1,38931	1,14439	1,17514	7,67138	1,50124	1,52504	1,63648	2,47982
SorghumBicolor	SB	10,49465	1,19527	0,43111	3,83105	3,68071	0,00000	5,94895	7,96210	6,63469	4,88431	2,54580	4,61885	11,35117	5,14897	2,30252	5,21410	5,92248
ZeaMays	ZM	4,63983	7,05654	5,62126	2,34349	2,42731	5,94895	0,00000	2,08951	0,89971	1,07070	3,44455	1,33187	5,57260	1,01544	3,64940	0,79480	0,25422
ArabidopsisThalianaCol	AT	2,55431	9,09760	7,65686	4,19212	4,31794	7,96210	2,08951	0,00000	1,32999	3,10061	5,42207	3,36875	3,48457	2,82045	5,68327	2,75348	2,20988
BrassicaRapaPekinensis	BRP	3,86560	7,77550	6,33426	2,86577	2,98890	6,63469	0,89971	1,32999	0,00000	1,79722	4,09292	2,06345	4,75981	1,49046	4,36299	1,43839	1,10720
CaricaPapaya	CP	5,65280	6,00313	4,56440	1,33369	1,38931	4,88431	1,07070	3,10061	1,79722	0,00000	2,37387	0,26817	6,55680	0,53151	2,58986	0,37005	1,09325
GlycineMax	GM	7,94947	3,70820	2,27378	1,30564	1,14439	2,54580	3,44455	5,42207	4,09292	2,37387	0,00000	2,11604	8,80681	2,60460	0,49175	2,68519	3,44771
ManihotEsculenta	ME	5,92075	5,73547	4,29708	1,14372	1,17514	4,61885	1,33187	3,36875	2,06345	0,26817	2,11604	0,00000	6,82326	0,72002	2,32275	0,62876	1,33579
PopulusTrichocarpa	PT	1,00890	12,51143	11,07140	7,52569	7,67138	11,35117	5,57260	3,48457	4,75981	6,55680	8,80681	6,82326	0,00000	6,21568	9,10975	6,19743	5,69228
VitisVinifera	VV	5,34579	6,29827	4,85732	1,38715	1,50124	5,14897	1,01544	2,82045	1,49046	0,53151	2,60460	0,72002	6,21568	0,00000	2,89461	0,31092	1,15574
SelaginellaMoellendorffii	SM	8,22858	3,41453	1,97469	1,69893	1,52504	2,30252	3,64940	5,68327	4,36299	2,58986	0,49175	2,32275	9,10975	2,89461	0,00000	2,92980	3,61998
PhyscomitrellaPatens	PP	5,30131	6,34422	4,90369	1,54885	1,63648	5,21410	0,79480	2,75348	1,43839	0,37005	2,68519	0,62876	6,19743	0,31092	2,92980	0,00000	0,88995
ChlamydomonasReinhardtii	CR	4,74334	7,01173	5,58287	2,41251	2,47982	5,92248	0,25422	2,20988	1,10720	1,09325	3,44771	1,33579	5,69228	1,15574	3,61998	0,88995	0,00000

Table S9: Distance matrix derived from the **betweenness** measure using the *commom-metabolites-set* **.** Each pair represent the distance formed among each pair of the 17 plants species.

		BD	HV	OSJ	PV	SI	SB	ZM	AT	BRP	CP	GM	ME	PT	VV	SM	PP	CR
BrachypodiumDistachyon	BD	0,00000	0,06885	0,01992	0,03936	0,05297	0,00454	0,06372	0,11933	0,07307	0,04731	0,12542	0,09797	0,09156	0,11979	0,05592	0,14204	1,26875
HordeumVulgare	HV	0,06885	0,00000	0,05366	0,05484	0,02633	0,06550	0,01576	0,05232	0,01184	0,03189	0,11686	0,03238	0,02271	0,05342	0,04620	0,20522	1,30691
OryzaSativaJaponica	OSJ	0,01992	0,05366	0,00000	0,04328	0,04451	0,01541	0,04581	0,10572	0,05578	0,02800	0,13081	0,08492	0,07606	0,10657	0,03626	0,15211	1,28711
PanicumVirgatum	PV	0,03936	0,05484	0,04328	0,00000	0,02909	0,03993	0,05950	0,09507	0,06449	0,05402	0,08776	0,07412	0,07449	0,09463	0,07093	0,17865	1,25560
SetariaItalica	SI	0,05297	0,02633	0,04451	0,02909	0,00000	0,05093	0,03465	0,06820	0,03705	0,03777	0,09711	0,04677	0,04544	0,06821	0,05606	0,19459	1,28112
SorghumBicolor	SB	0,00454	0,06550	0,01541	0,03993	0,05093	0,00000	0,05975	0,11646	0,06926	0,04299	0,12689	0,09519	0,08820	0,11700	0,05138	0,14368	1,27311
ZeaMays	ZM	0,06372	0,01576	0,04581	0,05950	0,03465	0,05975	0,00000	0,06451	0,01047	0,01943	0,13010	0,04636	0,03285	0,06607	0,03086	0,19448	1,31480
ArabidopsisThalianaCol	AT	0,11933	0,05232	0,10572	0,09507	0,06820	0,11646	0,06451	0,00000	0,05441	0,08338	0,11892	0,02148	0,03180	0,00311	0,09429	0,25753	1,32122
BrassicaRapaPekinensis	BRP	0,07307	0,01184	0,05578	0,06449	0,03705	0,06926	0,01047	0,05441	0,00000	0,02990	0,12863	0,03724	0,02262	0,05610	0,04009	0,20495	1,31814
CaricaPapaya	CP	0,04731	0,03189	0,02800	0,05402	0,03777	0,04299	0,01943	0,08338	0,02990	0,00000	0,13446	0,06420	0,05201	0,08476	0,01834	0,17506	1,30830
GlycineMax	GM	0,12542	0,11686	0,13081	0,08776	0,09711	0,12689	0,13010	0,11892	0,12863	0,13446	0,00000	0,10908	0,12239	0,11636	0,15278	0,25271	1,20237
ManihotEsculenta	ME	0,09797	0,03238	0,08492	0,07412	0,04677	0,09519	0,04636	0,02148	0,03724	0,06420	0,10908	0,00000	0,01724	0,02182	0,07710	0,23698	1,30935
PopulusTrichocarpa	PT	0,09156	0,02271	0,07606	0,07449	0,04544	0,08820	0,03285	0,03180	0,02262	0,05201	0,12239	0,01724	0,00000	0,03359	0,06252	0,22687	1,31979
VitisVinifera	VV	0,11979	0,05342	0,10657	0,09463	0,06821	0,11700	0,06607	0,00311	0,05610	0,08476	0,11636	0,02182	0,03359	0,00000	0,09610	0,25853	1,31871
SelaginellaMoellendorffii	SM	0,05592	0,04620	0,03626	0,07093	0,05606	0,05138	0,03086	0,09429	0,04009	0,01834	0,15278	0,07710	0,06252	0,09610	0,00000	0,16926	1,32266
PhyscomitrellaPatens	PP	0,14204	0,20522	0,15211	0,17865	0,19459	0,14368	0,19448	0,25753	0,20495	0,17506	0,25271	0,23698	0,22687	0,25853	0,16926	0,00000	1,26699
ChlamydomonasReinhardtii	CR	1,26875	1,30691	1,28711	1,25560	1,28112	1,27311	1,31480	1,32122	1,31814	1,30830	1,20237	1,30935	1,31979	1,31871	1,32266	1,26699	0,00000

Table S10: Distance matrix derived from the **eigen centrality** measure using the *commom-metabolites-set*. Each pair represent the distance formed among each pair of the 17 plants species.

		BD	HV	OSJ	PV	SI	SB	ZM	AT	BRP	CP	GM	ME	PT	VV	SM	PP	CR
BrachypodiumDistachyon	BD	0,00000	1,20367	0,06172	1,22246	1,21363	1,22211	1,18953	1,14788	1,17829	1,21486	1,23783	1,19717	1,17426	1,18740	1,22704	1,32994	0,98425
HordeumVulgare	HV	1,20367	0,00000	1,21361	0,14482	0,11969	0,12777	0,05758	0,05629	0,03547	0,06874	0,18663	0,04400	0,03165	0,01791	0,04223	0,29649	1,77568
OryzaSativaJaponica	OSJ	0,06172	1,21361	0,00000	1,23946	1,22948	1,23828	1,20230	1,15751	1,18952	1,22817	1,25669	1,20930	1,18364	1,19698	1,23870	1,35229	0,92358
PanicumVirgatum	PV	1,22246	0,14482	1,23946	0,00000	0,02559	0,01730	0,09261	0,16552	0,12518	0,07635	0,04227	0,10257	0,16096	0,15420	0,10968	0,16093	1,86419
SetariaItalica	SI	1,21363	0,11969	1,22948	0,02559	0,00000	0,01086	0,06704	0,14034	0,09963	0,05177	0,06786	0,07707	0,13537	0,12868	0,08619	0,18539	1,84484
SorghumBicolor	SB	1,22211	0,12777	1,23828	0,01730	0,01086	0,00000	0,07675	0,15058	0,10932	0,05917	0,05891	0,08598	0,14477	0,13757	0,09239	0,17496	1,85555
ZeaMays	ZM	1,18953	0,05758	1,20230	0,09261	0,06704	0,07675	0,00000	0,07465	0,03259	0,02776	0,13485	0,01458	0,06846	0,06292	0,04319	0,25117	1,79264
ArabidopsisThalianaCol	AT	1,14788	0,05629	1,15751	0,16552	0,14034	0,15058	0,07465	0,00000	0,04387	0,09963	0,20729	0,07018	0,02667	0,03951	0,08957	0,32548	1,72455
BrassicaRapaPekinensis	BRP	1,17829	0,03547	1,18952	0,12518	0,09963	0,10932	0,03259	0,04387	0,00000	0,05585	0,16741	0,02632	0,03626	0,03330	0,04972	0,28347	1,76715
CaricaPapaya	CP	1,21486	0,06874	1,22817	0,07635	0,05177	0,05917	0,02776	0,09963	0,05585	0,00000	0,11793	0,02976	0,08857	0,07977	0,03532	0,22945	1,82023
GlycineMax	GM	1,23783	0,18663	1,25669	0,04227	0,06786	0,05891	0,13485	0,20729	0,16741	0,11793	0,00000	0,14478	0,20323	0,19640	0,15006	0,12167	1,89630
ManihotEsculenta	ME	1,19717	0,04400	1,20930	0,10257	0,07707	0,08598	0,01458	0,07018	0,02632	0,02976	0,14478	0,00000	0,05923	0,05163	0,03116	0,25868	1,79281
PopulusTrichocarpa	PT	1,17426	0,03165	1,18364	0,16096	0,13537	0,14477	0,06846	0,02667	0,03626	0,08857	0,20323	0,05923	0,00000	0,01380	0,07036	0,31789	1,74457
VitisVinifera	VV	1,18740	0,01791	1,19698	0,15420	0,12868	0,13757	0,06292	0,03951	0,03330	0,07977	0,19640	0,05163	0,01380	0,00000	0,05812	0,30910	1,75791
SelaginellaMoellendorffii	SM	1,22704	0,04223	1,23870	0,10968	0,08619	0,09239	0,04319	0,08957	0,04972	0,03532	0,15006	0,03116	0,07036	0,05812	0,00000	0,25568	1,81391
PhyscomitrellaPatens	PP	1,32994	0,29649	1,35229	0,16093	0,18539	0,17496	0,25117	0,32548	0,28347	0,22945	0,12167	0,25868	0,31789	0,30910	0,25568	0,00000	2,01408
ChlamydomonasReinhardtii	CR	0,98425	1,77568	0,92358	1,86419	1,84484	1,85555	1,79264	1,72455	1,76715	1,82023	1,89630	1,79281	1,74457	1,75791	1,81391	2,01408	0,00000

Table S11: Distance matrix derived from the **hub-score** measure using the *full-metabolites-set* . Each pair represent the distance formed among each pair of the 17 plants species.

		BD	HV	OSJ	PV	SI	SB	ZM	AT	BRP	CP	GM	ME	PT	VV	SM	PP	CR
BrachypodiumDistachyon	BD	0,00000	0,01082	0,02869	0,03009	0,02786	0,01697	0,00808	0,07983	0,06815	0,09007	0,05385	0,09519	0,08551	0,07108	0,20180	0,28688	0,83999
HordeumVulgare	HV	0,01082	0,00000	0,02007	0,02097	0,01936	0,00949	0,00987	0,06901	0,05775	0,07983	0,04367	0,08586	0,07587	0,06130	0,21023	0,29507	0,84787
OryzaSativaJaponica	OSJ	0,02869	0,02007	0,00000	0,00229	0,00090	0,01173	0,02994	0,05690	0,04118	0,06233	0,02654	0,06651	0,05696	0,04271	0,23013	0,31505	0,86792
PanicumVirgatum	PV	0,03009	0,02097	0,00229	0,00000	0,00309	0,01324	0,03081	0,05464	0,03917	0,06049	0,02455	0,06513	0,05543	0,04106	0,23119	0,31604	0,86882
SetariaItalica	SI	0,02786	0,01936	0,00090	0,00309	0,00000	0,01089	0,02923	0,05772	0,04208	0,06322	0,02744	0,06735	0,05784	0,04360	0,22935	0,31429	0,86718
SorghumBicolor	SB	0,01697	0,00949	0,01173	0,01324	0,01089	0,00000	0,01904	0,06589	0,05207	0,07364	0,03753	0,07823	0,06864	0,05430	0,21859	0,30358	0,85657
ZeaMays	ZM	0,00808	0,00987	0,02994	0,03081	0,02923	0,01904	0,00000	0,07622	0,06662	0,08884	0,05286	0,09549	0,08537	0,07075	0,20045	0,28524	0,83801
ArabidopsisThalianaCol	AT	0,07983	0,06901	0,05690	0,05464	0,05772	0,06589	0,07622	0,00000	0,02243	0,02836	0,03301	0,04619	0,03692	0,03198	0,26794	0,35052	0,89941
BrassicaRapaPekinensis	BRP	0,06815	0,05775	0,04118	0,03917	0,04208	0,05207	0,06662	0,02243	0,00000	0,02230	0,01464	0,03342	0,02235	0,01089	0,26512	0,34907	0,90033
CaricaPapaya	CP	0,09007	0,07983	0,06233	0,06049	0,06322	0,07364	0,08884	0,02836	0,02230	0,00000	0,03621	0,01846	0,01244	0,02054	0,28729	0,37109	0,92195
GlycineMax	GM	0,05385	0,04367	0,02654	0,02455	0,02744	0,03753	0,05286	0,03301	0,01464	0,03621	0,00000	0,04358	0,03292	0,01829	0,25251	0,33684	0,88878
ManihotEsculenta	ME	0,09519	0,08586	0,06651	0,06513	0,06735	0,07823	0,09549	0,04619	0,03342	0,01846	0,04358	0,00000	0,01108	0,02531	0,29579	0,38030	0,93236
PopulusTrichocarpa	PT	0,08551	0,07587	0,05696	0,05543	0,05784	0,06864	0,08537	0,03692	0,02235	0,01244	0,03292	0,01108	0,00000	0,01467	0,28541	0,36977	0,92162
VitisVinifera	VV	0,07108	0,06130	0,04271	0,04106	0,04360	0,05430	0,07075	0,03198	0,01089	0,02054	0,01829	0,02531	0,01467	0,00000	0,27074	0,35512	0,90706
SelaginellaMoellendorffii	SM	0,20180	0,21023	0,23013	0,23119	0,22935	0,21859	0,20045	0,26794	0,26512	0,28729	0,25251	0,29579	0,28541	0,27074	0,00000	0,08539	0,63875
PhyscomitrellaPatens	PP	0,28688	0,29507	0,31505	0,31604	0,31429	0,30358	0,28524	0,35052	0,34907	0,37109	0,33684	0,38030	0,36977	0,35512	0,08539	0,00000	0,55338
ChlamydomonasReinhardtii	CR	0,83999	0,84787	0,86792	0,86882	0,86718	0,85657	0,83801	0,89941	0,90033	0,92195	0,88878	0,93236	0,92162	0,90706	0,63875	0,55338	0,00000

Table S12: Distance matrix derived from the **clustering coefficient** measure using the *full-metabolites-set*. Each pair represent the distance formed among each pair of the 17 plants species.

		BD	HV	OSJ	PV	SI	SB	ZM	AT	BRP	CP	GM	ME	PT	VV	SM	PP	CR
BrachypodiumDistachyon	BD	0,00000	2,85269	2,72722	3,47904	3,08117	2,53744	2,87762	7,08797	3,45180	1,06614	0,02660	1,56543	3,77742	1,79084	2,39971	1,66489	3,55811
HordeumVulgare	HV	2,85269	0,00000	0,28552	0,72834	0,44116	0,40570	0,18799	9,93858	6,30420	3,91815	2,87015	4,41609	6,62575	4,62545	0,93212	2,17171	4,80164
OryzaSativaJaponica	OSJ	2,72722	0,28552	0,00000	0,75610	0,36852	0,18994	0,46448	9,81304	6,16956	3,79240	2,74645	4,28065	6,48302	4,47774	1,07897	2,22398	4,90090
PanicumVirgatum	PV	3,47904	0,72834	0,75610	0,00000	0,39904	0,94410	0,83453	10,56083	6,91446	4,54282	3,49873	5,02672	7,22333	5,21701	1,64007	2,89991	5,51328
SetariaItalica	SI	3,08117	0,44116	0,36852	0,39904	0,00000	0,55124	0,60722	10,16190	6,51542	4,14442	3,10103	4,62773	6,82445	4,81816	1,36857	2,57263	5,22883
SorghumBicolor	SB	2,53744	0,40570	0,18994	0,94410	0,55124	0,00000	0,54976	9,62311	5,97967	3,60253	2,55673	4,09074	6,29339	4,28827	1,00826	2,08174	4,77486
ZeaMays	ZM	2,87762	0,18799	0,46448	0,83453	0,60722	0,54976	0,00000	9,95315	6,32779	3,93930	2,89374	4,44305	6,65499	4,66100	0,80594	2,08471	4,67875
ArabidopsisThalianaCol	AT	7,08797	9,93858	9,81304	10,56083	10,16190	9,62311	9,95315	0,00000	3,65024	6,02184	7,06972	5,53438	3,36685	5,35805	9,37340	8,22036	7,41807
BrassicaRapaPekinensis	BRP	3,45180	6,30420	6,16956	6,91446	6,51542	5,97967	6,32779	3,65024	0,00000	2,38858	3,43487	1,88913	0,37918	1,70892	5,79081	4,72751	4,72436
CaricaPapaya	CP	1,06614	3,91815	3,79240	4,54282	4,14442	3,60253	3,93930	6,02184	2,38858	0,00000	1,04810	0,51752	2,72113	0,78744	3,41961	2,47145	3,55487
GlycineMax	GM	0,02660	2,87015	2,74645	3,49873	3,10103	2,55673	2,89374	7,06972	3,43487	1,04810	0,00000	1,54950	3,76161	1,77752	2,41019	1,66117	3,53620
ManihotEsculenta	ME	1,56543	4,41609	4,28065	5,02672	4,62773	4,09074	4,44305	5,53438	1,88913	0,51752	1,54950	0,00000	2,21212	0,29961	3,93577	2,97737	3,81784
PopulusTrichocarpa	PT	3,77742	6,62575	6,48302	7,22333	6,82445	6,29339	6,65499	3,36685	0,37918	2,72113	3,76161	2,21212	0,00000	2,00634	6,13517	5,09100	5,08960
VitisVinifera	VV	1,79084	4,62545	4,47774	5,21701	4,81816	4,28827	4,66100	5,35805	1,70892	0,78744	1,77752	0,29961	2,00634	0,00000	4,18172	3,25864	4,09360
SelaginellaMoellendorffii	SM	2,39971	0,93212	1,07897	1,64007	1,36857	1,00826	0,80594	9,37340	5,79081	3,41961	2,41019	3,93577	6,13517	4,18172	0,00000	1,30756	3,87379
PhyscomitrellaPatens	PP	1,66489	2,17171	2,22398	2,89991	2,57263	2,08174	2,08471	8,22036	4,72751	2,47145	1,66117	2,97737	5,09100	3,25864	1,30756	0,00000	2,71365
ChlamydomonasReinhardtii	CR	3,55811	4,80164	4,90090	5,51328	5,22883	4,77486	4,67875	7,41807	4,72436	3,55487	3,53620	3,81784	5,08960	4,09360	3,87379	2,71365	0,00000

Table S13: Distance matrix derived from the **degree** measure using the *full-metabolites-set* . Each pair represent the distance formed among each pair of the 17 plants species.

		BD	HV	OSJ	PV	SI	SB	ZM	AT	BRP	CP	GM	ME	PT	VV	SM	PP	CR
BrachypodiumDistachyon	BD	0,00000	0,01721	0,02651	0,04110	0,02531	0,01106	0,01000	0,13816	0,05114	0,03316	0,07613	0,07574	0,10340	0,06593	0,07513	0,10272	0,33892
HordeumVulgare	HV	0,01721	0,00000	0,00956	0,02391	0,00914	0,01004	0,00833	0,12109	0,03395	0,01613	0,05923	0,05855	0,08620	0,04874	0,09080	0,11698	0,35582
OryzaSativaJaponica	OSJ	0,02651	0,00956	0,00000	0,01474	0,00287	0,01747	0,01688	0,11244	0,02479	0,00665	0,05105	0,04930	0,07721	0,03987	0,10032	0,12624	0,36533
PanicumVirgatum	PV	0,04110	0,02391	0,01474	0,00000	0,01667	0,03216	0,03161	0,09776	0,01006	0,00825	0,03682	0,03464	0,06247	0,02516	0,11376	0,13859	0,37958
SetariaItalica	SI	0,02531	0,00914	0,00287	0,01667	0,00000	0,01558	0,01542	0,11442	0,02661	0,00842	0,05331	0,05091	0,07902	0,04182	0,09976	0,12612	0,36423
SorghumBicolor	SB	0,01106	0,01004	0,01747	0,03216	0,01558	0,00000	0,00296	0,12990	0,04216	0,02393	0,06844	0,06649	0,09459	0,05731	0,08613	0,11376	0,34918
ZeaMays	ZM	0,01000	0,00833	0,01688	0,03161	0,01542	0,00296	0,00000	0,12917	0,04167	0,02350	0,06744	0,06617	0,09408	0,05668	0,08503	0,11226	0,34885
ArabidopsisThalianaCol	AT	0,13816	0,12109	0,11244	0,09776	0,11442	0,12990	0,12917	0,00000	0,08785	0,10601	0,06222	0,06455	0,03620	0,07261	0,20496	0,22428	0,47235
BrassicaRapaPekinensis	BRP	0,05114	0,03395	0,02479	0,01006	0,02661	0,04216	0,04167	0,08785	0,00000	0,01823	0,02770	0,02461	0,05243	0,01528	0,12329	0,14754	0,38945
CaricaPapaya	CP	0,03316	0,01613	0,00665	0,00825	0,00842	0,02393	0,02350	0,10601	0,01823	0,00000	0,04496	0,04267	0,07066	0,03341	0,10671	0,13227	0,37195
GlycineMax	GM	0,07613	0,05923	0,05105	0,03682	0,05331	0,06844	0,06744	0,06222	0,02770	0,04496	0,00000	0,01582	0,02969	0,01351	0,14369	0,16494	0,41107
ManihotEsculenta	ME	0,07574	0,05855	0,04930	0,03464	0,05091	0,06649	0,06617	0,06455	0,02461	0,04267	0,01582	0,00000	0,02847	0,01145	0,14744	0,17080	0,41396
PopulusTrichocarpa	PT	0,10340	0,08620	0,07721	0,06247	0,07902	0,09459	0,09408	0,03620	0,05243	0,07066	0,02969	0,02847	0,00000	0,03748	0,17305	0,19463	0,44029
VitisVinifera	VV	0,06593	0,04874	0,03987	0,02516	0,04182	0,05731	0,05668	0,07261	0,01528	0,03341	0,01351	0,01145	0,03748	0,00000	0,13651	0,15951	0,40334
SelaginellaMoellendorffii	SM	0,07513	0,09080	0,10032	0,11376	0,09976	0,08613	0,08503	0,20496	0,12329	0,10671	0,14369	0,14744	0,17305	0,13651	0,00000	0,03126	0,26744
PhyscomitrellaPatens	PP	0,10272	0,11698	0,12624	0,13859	0,12612	0,11376	0,11226	0,22428	0,14754	0,13227	0,16494	0,17080	0,19463	0,15951	0,03126	0,00000	0,25011
ChlamydomonasReinhardtii	CR	0,33892	0,35582	0,36533	0,37958	0,36423	0,34918	0,34885	0,47235	0,38945	0,37195	0,41107	0,41396	0,44029	0,40334	0,26744	0,25011	0,00000

Table S14: Distance matrix derived from the **in-degree** measure using the ***full-metabolites-set*** **.** Each pair represent the distance formed among each pair of the 17 plants species.

		BD	HV	OSJ	PV	SI	SB	ZM	AT	BRP	CP	GM	ME	PT	VV	SM	PP	CR
BrachypodiumDistachyon	BD	0,00000	0,01556	0,02618	0,03753	0,02027	0,01138	0,00966	0,14162	0,05822	0,04058	0,07391	0,07573	0,10154	0,06573	0,06978	0,09610	0,32006
HordeumVulgare	HV	0,01556	0,00000	0,01079	0,02218	0,00770	0,00803	0,00607	0,12610	0,04330	0,02534	0,05841	0,06082	0,08618	0,05049	0,08223	0,10694	0,33438
OryzaSativaJaponica	OSJ	0,02618	0,01079	0,00000	0,01140	0,00824	0,01650	0,01652	0,11545	0,03258	0,01456	0,04773	0,05008	0,07541	0,03970	0,09243	0,11645	0,34504
PanicumVirgatum	PV	0,03753	0,02218	0,01140	0,00000	0,01826	0,02736	0,02787	0,10418	0,02142	0,00332	0,03646	0,03883	0,06402	0,02831	0,10324	0,12658	0,35625
SetariaItalica	SI	0,02027	0,00770	0,00824	0,01826	0,00000	0,00928	0,01109	0,12225	0,03810	0,02098	0,05459	0,05557	0,08175	0,04586	0,08902	0,11420	0,34029
SorghumBicolor	SB	0,01138	0,00803	0,01650	0,02736	0,00928	0,00000	0,00457	0,13148	0,04737	0,03019	0,06379	0,06481	0,09102	0,05514	0,08100	0,10692	0,33138
ZeaMays	ZM	0,00966	0,00607	0,01652	0,02787	0,01109	0,00457	0,00000	0,13197	0,04867	0,03094	0,06425	0,06619	0,09188	0,05609	0,07797	0,10342	0,32931
ArabidopsisThalianaCol	AT	0,14162	0,12610	0,11545	0,10418	0,12225	0,13148	0,13197	0,00000	0,08494	0,10128	0,06773	0,06874	0,04123	0,07662	0,20195	0,22019	0,45596
BrassicaRapaPekinensis	BRP	0,05822	0,04330	0,03258	0,02142	0,03810	0,04737	0,04867	0,08494	0,00000	0,01812	0,01906	0,01752	0,04395	0,00861	0,12464	0,14763	0,37762
CaricaPapaya	CP	0,04058	0,02534	0,01456	0,00332	0,02098	0,03019	0,03094	0,10128	0,01812	0,00000	0,03361	0,03556	0,06096	0,02516	0,10655	0,12988	0,35952
GlycineMax	GM	0,07391	0,05841	0,04773	0,03646	0,05459	0,06379	0,06425	0,06773	0,01906	0,03361	0,00000	0,01337	0,02825	0,01061	0,13668	0,15760	0,39072
ManihotEsculenta	ME	0,07573	0,06082	0,05008	0,03883	0,05557	0,06481	0,06619	0,06874	0,01752	0,03556	0,01337	0,00000	0,02752	0,01149	0,14185	0,16430	0,39508
PopulusTrichocarpa	PT	0,10154	0,08618	0,07541	0,06402	0,08175	0,09102	0,09188	0,04123	0,04395	0,06096	0,02825	0,02752	0,00000	0,03589	0,16490	0,18540	0,41896
VitisVinifera	VV	0,06573	0,05049	0,03970	0,02831	0,04586	0,05514	0,05609	0,07662	0,00861	0,02516	0,01061	0,01149	0,03589	0,00000	0,13078	0,15293	0,38432
SelaginellaMoellendorffii	SM	0,06978	0,08223	0,09243	0,10324	0,08902	0,08100	0,07797	0,20195	0,12464	0,10655	0,13668	0,14185	0,16490	0,13078	0,00000	0,02899	0,25417
PhyscomitrellaPatens	PP	0,09610	0,10694	0,11645	0,12658	0,11420	0,10692	0,10342	0,22019	0,14763	0,12988	0,15760	0,16430	0,18540	0,15293	0,02899	0,00000	0,23703
ChlamydomonasReinhardtii	CR	0,32006	0,33438	0,34504	0,35625	0,34029	0,33138	0,32931	0,45596	0,37762	0,35952	0,39072	0,39508	0,41896	0,38432	0,25417	0,23703	0,00000

Table S15: Distance matrix derived from the **out-degree** measure using the *full-metabolites-set*. Each pair represent the distance formed among each pair of the 17 plants species.

		BD	HV	OSJ	PV	SI	SB	ZM	AT	BRP	CP	GM	ME	PT	VV	SM	PP	CR
BrachypodiumDistachyon	BD	0,00000	0,01694	0,03149	0,04412	0,02978	0,00953	0,02148	0,14469	0,04821	0,02934	0,08513	0,07792	0,10817	0,06717	0,10220	0,13097	0,39051
HordeumVulgare	HV	0,01694	0,00000	0,01850	0,02779	0,01469	0,01338	0,01831	0,12791	0,03196	0,01629	0,06826	0,06142	0,09148	0,05033	0,11565	0,14308	0,40623
OryzaSativaJaponica	OSJ	0,03149	0,01850	0,00000	0,01655	0,00578	0,02305	0,01565	0,11589	0,01969	0,00224	0,05780	0,04867	0,07922	0,04018	0,13320	0,16118	0,42183
PanicumVirgatum	PV	0,04412	0,02779	0,01655	0,00000	0,01486	0,03731	0,03205	0,10077	0,00418	0,01760	0,04178	0,03380	0,06416	0,02390	0,14265	0,16911	0,43401
SetariaItalica	SI	0,02978	0,01469	0,00578	0,01486	0,00000	0,02248	0,01810	0,11557	0,01872	0,00471	0,05663	0,04846	0,07893	0,03870	0,13030	0,15776	0,42022
SorghumBicolor	SB	0,00953	0,01338	0,02305	0,03731	0,02248	0,00000	0,01198	0,13805	0,04120	0,02105	0,07898	0,07091	0,10140	0,06100	0,11155	0,14046	0,39883
ZeaMays	ZM	0,02148	0,01831	0,01565	0,03205	0,01810	0,01198	0,00000	0,13126	0,03533	0,01446	0,07344	0,06413	0,09463	0,05581	0,12306	0,15224	0,40869
ArabidopsisThalianaCol	AT	0,14469	0,12791	0,11589	0,10077	0,11557	0,13805	0,13126	0,00000	0,09686	0,11753	0,05981	0,06723	0,03667	0,07760	0,23717	0,25955	0,53164
BrassicaRapaPekinensis	BRP	0,04821	0,03196	0,01969	0,00418	0,01872	0,04120	0,03533	0,09686	0,00000	0,02100	0,03817	0,02976	0,06021	0,02049	0,14681	0,17319	0,43818
CaricaPapaya	CP	0,02934	0,01629	0,00224	0,01760	0,00471	0,02105	0,01446	0,11753	0,02100	0,00000	0,05917	0,05031	0,08086	0,04143	0,13097	0,15893	0,41975
GlycineMax	GM	0,08513	0,06826	0,05780	0,04178	0,05663	0,07898	0,07344	0,05981	0,03817	0,05917	0,00000	0,01235	0,02419	0,01800	0,17845	0,20226	0,47231
ManihotEsculenta	ME	0,07792	0,06142	0,04867	0,03380	0,04846	0,07091	0,06413	0,06723	0,02976	0,05031	0,01235	0,00000	0,03055	0,01332	0,17467	0,19976	0,46731
PopulusTrichocarpa	PT	0,10817	0,09148	0,07922	0,06416	0,07893	0,10140	0,09463	0,03667	0,06021	0,08086	0,02419	0,03055	0,00000	0,04135	0,20264	0,22634	0,49640
VitisVinifera	VV	0,06717	0,05033	0,04018	0,02390	0,03870	0,06100	0,05581	0,07760	0,02049	0,04143	0,01800	0,01332	0,04135	0,00000	0,16198	0,18671	0,45520
SelaginellaMoellendorffii	SM	0,10220	0,11565	0,13320	0,14265	0,13030	0,11155	0,12306	0,23717	0,14681	0,13097	0,17845	0,17467	0,20264	0,16198	0,00000	0,03175	0,29491
PhyscomitrellaPatens	PP	0,13097	0,14308	0,16118	0,16911	0,15776	0,14046	0,15224	0,25955	0,17319	0,15893	0,20226	0,19976	0,22634	0,18671	0,03175	0,00000	0,27674
ChlamydomonasReinhardtii	CR	0,39051	0,40623	0,42183	0,43401	0,42022	0,39883	0,40869	0,53164	0,43818	0,41975	0,47231	0,46731	0,49640	0,45520	0,29491	0,27674	0,00000

Table S16: Distance matrix derived from the **authority-score** measure using the *full-metabolites-set* **.** Each pair represent the distance formed among each pair of the 17 plants species.

		BD	HV	OSJ	PV	SI	SB	ZM	AT	BRP	CP	GM	ME	PT	VV	SM	PP	CR
BrachypodiumDistachyon	BD	0,00000	0,01339	0,01105	0,01362	0,01977	0,00474	0,01419	0,02337	0,01441	0,01234	0,03005	0,03148	0,03434	0,02247	0,06505	0,11474	0,35606
HordeumVulgare	HV	0,01339	0,00000	0,00315	0,00699	0,00867	0,00939	0,02002	0,01519	0,00401	0,00825	0,01865	0,01814	0,02110	0,01018	0,07820	0,12746	0,36845
OryzaSativaJaponica	OSJ	0,01105	0,00315	0,00000	0,00876	0,01172	0,00665	0,01985	0,01791	0,00660	0,00563	0,02180	0,02054	0,02332	0,01333	0,07609	0,12566	0,36684
PanicumVirgatum	PV	0,01362	0,00699	0,00876	0,00000	0,00669	0,01170	0,01456	0,01005	0,00348	0,01438	0,01656	0,02150	0,02476	0,00991	0,07573	0,12409	0,36449
SetariaItalica	SI	0,01977	0,00867	0,01172	0,00669	0,00000	0,01692	0,02054	0,00730	0,00554	0,01684	0,01038	0,01575	0,01907	0,00336	0,08237	0,13053	0,37067
SorghumBicolor	SB	0,00474	0,00939	0,00665	0,01170	0,01692	0,00000	0,01704	0,02174	0,01138	0,00772	0,02730	0,02718	0,02994	0,01920	0,06965	0,11944	0,36079
ZeaMays	ZM	0,01419	0,02002	0,01985	0,01456	0,02054	0,01704	0,00000	0,01924	0,01780	0,02407	0,02826	0,03601	0,03929	0,02390	0,06287	0,11017	0,35013
ArabidopsisThalianaCol	AT	0,02337	0,01519	0,01791	0,01005	0,00730	0,02174	0,01924	0,00000	0,01131	0,02341	0,00918	0,02081	0,02401	0,00897	0,08211	0,12901	0,36816
BrassicaRapaPekinensis	BRP	0,01441	0,00401	0,00660	0,00348	0,00554	0,01138	0,01780	0,01131	0,00000	0,01211	0,01592	0,01857	0,02176	0,00806	0,07805	0,12679	0,36741
CaricaPapaya	CP	0,01234	0,00825	0,00563	0,01438	0,01684	0,00772	0,02407	0,02341	0,01211	0,00000	0,02643	0,02226	0,02454	0,01785	0,07640	0,12653	0,36802
GlycineMax	GM	0,03005	0,01865	0,02180	0,01656	0,01038	0,02730	0,02826	0,00918	0,01592	0,02643	0,00000	0,01481	0,01751	0,00858	0,09107	0,13819	0,37728
ManihotEsculenta	ME	0,03148	0,01814	0,02054	0,02150	0,01575	0,02718	0,03601	0,02081	0,01857	0,02226	0,01481	0,00000	0,00331	0,01256	0,09631	0,14532	0,38596
PopulusTrichocarpa	PT	0,03434	0,02110	0,02332	0,02476	0,01907	0,02994	0,03929	0,02401	0,02176	0,02454	0,01751	0,00331	0,00000	0,01587	0,09929	0,14845	0,38917
VitisVinifera	VV	0,02247	0,01018	0,01333	0,00991	0,00336	0,01920	0,02390	0,00897	0,00806	0,01785	0,00858	0,01256	0,01587	0,00000	0,08564	0,13388	0,37403
SelaginellaMoellendorffii	SM	0,06505	0,07820	0,07609	0,07573	0,08237	0,06965	0,06287	0,08211	0,07805	0,07640	0,09107	0,09631	0,09929	0,08564	0,00000	0,05081	0,29237
PhyscomitrellaPatens	PP	0,11474	0,12746	0,12566	0,12409	0,13053	0,11944	0,11017	0,12901	0,12679	0,12653	0,13819	0,14532	0,14845	0,13388	0,05081	0,00000	0,24165
ChlamydomonasReinhardtii	CR	0,35606	0,36845	0,36684	0,36449	0,37067	0,36079	0,35013	0,36816	0,36741	0,36802	0,37728	0,38596	0,38917	0,37403	0,29237	0,24165	0,00000

Table S17: Distance matrix derived from the **local efficiency** measure using the *full-metabolites-set* **.** Each pair represent the distance formed among each pair of the 17 plants species.

		BD	HV	OSJ	PV	SI	SB	ZM	AT	BRP	CP	GM	ME	PT	VV	SM	PP	CR
BrachypodiumDistachyon	BD	0,00000	3,79202	4,93402	5,28745	4,47440	3,60962	3,28739	12,20650	5,83392	2,33152	0,49052	1,56861	5,75481	2,80250	3,60757	2,45529	5,50327
HordeumVulgare	HV	3,79202	0,00000	1,19105	1,58960	0,89623	0,47299	0,60400	15,99848	9,62271	6,12329	3,58408	5,31855	9,53331	6,55467	1,06389	2,97414	7,02118
OryzaSativaJaponica	OSJ	4,93402	1,19105	0,00000	0,41400	0,54653	1,33500	1,78649	17,12832	10,74492	7,25813	4,68519	6,42842	10,64242	7,65587	2,01807	4,10594	8,05334
PanicumVirgatum	PV	5,28745	1,58960	0,41400	0,00000	0,81929	1,67786	2,19016	17,46489	11,07966	7,60335	5,01934	6,76171	10,96996	7,98256	2,42971	4,51947	8,46189
SetariaItalica	SI	4,47440	0,89623	0,54653	0,81929	0,00000	0,86976	1,48964	16,64590	10,26059	6,78637	4,20061	5,94269	10,15069	7,16329	1,90497	3,86843	7,91224
SorghumBicolor	SB	3,60962	0,47299	1,33500	1,67786	0,86976	0,00000	0,79718	15,79502	9,41094	5,92793	3,35055	5,09386	9,30746	6,32087	1,44186	3,12478	7,23483
ZeaMays	ZM	3,28739	0,60400	1,78649	2,19016	1,48964	0,79718	0,00000	15,48365	9,11955	5,61503	3,12766	4,83849	9,04197	6,07741	0,72819	2,38071	6,45862
ArabidopsisThalianaCol	AT	12,20650	15,99848	17,12832	17,46489	16,64590	15,79502	15,48365	0,00000	6,38541	9,87524	12,44577	10,70321	6,50988	9,48927	15,70591	13,87390	12,84739
BrassicaRapaPekinensis	BRP	5,83392	9,62271	10,74492	11,07966	10,26059	9,41094	9,11955	6,38541	0,00000	3,50471	6,06090	4,31797	0,33227	3,10773	9,38200	7,68228	7,78391
CaricaPapaya	CP	2,33152	6,12329	7,25813	7,60335	6,78637	5,92793	5,61503	9,87524	3,50471	0,00000	2,59423	0,90696	3,43917	0,70191	5,88920	4,32887	5,77481
GlycineMax	GM	0,49052	3,58408	4,68519	5,01934	4,20061	3,35055	3,12766	12,44577	6,06090	2,59423	0,00000	1,74332	5,95791	2,97327	3,53654	2,68611	5,96055
ManihotEsculenta	ME	1,56861	5,31855	6,42842	6,76171	5,94269	5,09386	4,83849	10,70321	4,31797	0,90696	1,74332	0,00000	4,21608	1,23893	5,17614	3,83863	5,96443
PopulusTrichocarpa	PT	5,75481	9,53331	10,64242	10,96996	10,15069	9,30746	9,04197	6,50988	0,33227	3,43917	5,95791	4,21608	0,00000	2,98741	9,32826	7,68077	7,95293
VitisVinifera	VV	2,80250	6,55467	7,65587	7,98256	7,16329	6,32087	6,07741	9,48927	3,10773	0,70191	2,97327	1,23893	2,98741	0,00000	6,40707	4,95962	6,44725
SelaginellaMoellendorffii	SM	3,60757	1,06389	2,01807	2,42971	1,90497	1,44186	0,72819	15,70591	9,38200	5,88920	3,53654	5,17614	9,32826	6,40707	0,00000	2,14249	6,03853
PhyscomitrellaPatens	PP	2,45529	2,97414	4,10594	4,51947	3,86843	3,12478	2,38071	13,87390	7,68228	4,32887	2,68611	3,83863	7,68077	4,95962	2,14249	0,00000	4,13811
ChlamydomonasReinhardtii	CR	5,50327	7,02118	8,05334	8,46189	7,91224	7,23483	6,45862	12,84739	7,78391	5,77481	5,96055	5,96443	7,95293	6,44725	6,03853	4,13811	0,00000

Table S18: Distance matrix derived from the **betweenness** measure using the *full-metabolites-set* **.** Each pair represent the distance formed among each pair of the 17 plants species.

		BD	HV	OSJ	PV	SI	SB	ZM	AT	BRP	CP	GM	ME	PT	VV	SM	PP	CR
BrachypodiumDistachyon	BD	0,00000	1,60761	0,59558	0,52326	0,53699	1,55727	0,55338	1,18786	1,58018	1,54477	0,60131	0,01172	0,00967	0,83053	1,64114	1,17404	1,13955
HordeumVulgare	HV	1,60761	0,00000	1,18065	1,29177	1,26683	0,07779	1,31497	1,07737	0,03189	0,07705	1,18299	1,61923	1,61510	0,98910	0,03353	1,23977	1,24747
OryzaSativaJaponica	OSJ	0,59558	1,18065	0,00000	0,11211	0,08717	1,15114	0,13706	0,59852	1,14961	1,13471	0,00907	0,60589	0,59807	0,24443	1,21255	0,61240	0,58171
PanicumVirgatum	PV	0,52326	1,29177	0,11211	0,00000	0,02499	1,26119	0,04994	0,66582	1,26081	1,24493	0,11141	0,53260	0,52430	0,34832	1,32378	0,65437	0,62085
SetariaItalica	SI	0,53699	1,26683	0,08717	0,02499	0,00000	1,23639	0,06394	0,65093	1,23587	1,22011	0,08673	0,54660	0,53841	0,32524	1,29884	0,64494	0,61195
SorghumBicolor	SB	1,55727	0,07779	1,15114	1,26119	1,23639	0,00000	1,28683	1,08859	0,07823	0,01897	1,15401	1,56883	1,56499	0,97055	0,10336	1,24913	1,25462
ZeaMays	ZM	0,55338	1,31497	0,13706	0,04994	0,06394	1,28683	0,00000	0,64252	1,28377	1,27025	0,13321	0,56217	0,55370	0,35285	1,34663	0,62075	0,58646
ArabidopsisThalianaCol	AT	1,18786	1,07737	0,59852	0,66582	0,65093	1,08859	0,64252	0,00000	1,04697	1,06963	0,59154	1,19752	1,18934	0,40794	1,09988	0,16396	0,17900
BrassicaRapaPekinensis	BRP	1,58018	0,03189	1,14961	1,26081	1,23587	0,07823	1,28377	1,04697	0,00000	0,06979	1,15189	1,59182	1,58761	0,95733	0,06301	1,20917	1,21663
CaricaPapaya	CP	1,54477	0,07705	1,13471	1,24493	1,22011	0,01897	1,27025	1,06963	0,06979	0,00000	1,13751	1,55634	1,55243	0,95284	0,10659	1,23016	1,23566
GlycineMax	GM	0,60131	1,18299	0,00907	0,11141	0,08673	1,15401	0,13321	0,59154	1,15189	1,13751	0,00000	0,61155	0,60369	0,24150	1,21481	0,60411	0,57331
ManihotEsculenta	ME	0,01172	1,61923	0,60589	0,53260	0,54660	1,56883	0,56217	1,19752	1,59182	1,55634	0,61155	0,00000	0,00870	0,84131	1,65276	1,18270	1,14814
PopulusTrichocarpa	PT	0,00967	1,61510	0,59807	0,52430	0,53841	1,56499	0,55370	1,18934	1,58761	1,55243	0,60369	0,00870	0,00000	0,83388	1,64863	1,17419	1,13961
VitisVinifera	VV	0,83053	0,98910	0,24443	0,34832	0,32524	0,97055	0,35285	0,40794	0,95733	0,95284	0,24150	0,84131	0,83388	0,00000	1,01928	0,47711	0,45623
SelaginellaMoellendorffii	SM	1,64114	0,03353	1,21255	1,32378	1,29884	0,10336	1,34663	1,09988	0,06301	0,10659	1,21481	1,65276	1,64863	1,01928	0,00000	1,26271	1,27110
PhyscomitrellaPatens	PP	1,17404	1,23977	0,61240	0,65437	0,64494	1,24913	0,62075	0,16396	1,20917	1,23016	0,60411	1,18270	1,17419	0,47711	1,26271	0,00000	0,03631
ChlamydomonasReinhardtii	CR	1,13955	1,24747	0,58171	0,62085	0,61195	1,25462	0,58646	0,17900	1,21663	1,23566	0,57331	1,14814	1,13961	0,45623	1,27110	0,03631	0,00000

Table S19: Distance matrix derived from the **eigen centrality** measure using the *full-metabolites-set* **.** Each pair represent the distance formed among each pair of the 17 plants species.

		BD	HV	OSJ	PV	SI	SB	ZM	AT	BRP	CP	GM	ME	PT	VV	SM	PP	CR
BrachypodiumDistachyon	BD	0,00000	1,59213	0,02451	1,57290	1,59262	1,68317	1,47989	1,26791	1,61394	1,64797	1,66484	1,69807	1,68997	1,55951	1,56271	1,27644	0,50037
HordeumVulgare	HV	1,59213	0,00000	1,57388	0,02616	0,00141	0,10982	0,14296	0,45286	0,02234	0,06386	0,08316	0,12311	0,11113	0,04770	0,03221	0,40528	1,18731
OryzaSativaJaponica	OSJ	0,02451	1,57388	0,00000	1,55483	1,57435	1,66430	1,46262	1,25378	1,59564	1,62941	1,64618	1,67920	1,67119	1,54163	1,54459	1,26143	0,47636
PanicumVirgatum	PV	1,57290	0,02616	1,55483	0,00000	0,02742	0,13581	0,11696	0,42678	0,04688	0,08956	0,10884	0,14889	0,13673	0,02167	0,01114	0,37942	1,17236
SetariaItalica	SI	1,59262	0,00141	1,57435	0,02742	0,00000	0,10867	0,14414	0,45407	0,02161	0,06279	0,08208	0,12200	0,11006	0,04899	0,03321	0,40642	1,18751
SorghumBicolor	SB	1,68317	0,10982	1,66430	0,13581	0,10867	0,00000	0,25276	0,56255	0,08960	0,04666	0,02812	0,01494	0,01135	0,15689	0,14155	0,51508	1,26427
ZeaMays	ZM	1,47989	0,14296	1,46262	0,11696	0,14414	0,25276	0,00000	0,30998	0,16361	0,20649	0,22574	0,26582	0,25357	0,09626	0,11178	0,26255	1,09868
ArabidopsisThalianaCol	AT	1,26791	0,45286	1,25378	0,42678	0,45407	0,56255	0,30998	0,00000	0,47309	0,51608	0,53525	0,57534	0,56289	0,40568	0,42175	0,05722	0,96503
BrassicaRapaPekinensis	BRP	1,61394	0,02234	1,59564	0,04688	0,02161	0,08960	0,16361	0,47309	0,00000	0,04300	0,06217	0,10227	0,08996	0,06744	0,05430	0,42615	1,20762
CaricaPapaya	CP	1,64797	0,06386	1,62941	0,08956	0,06279	0,04666	0,20649	0,51608	0,04300	0,00000	0,01930	0,05934	0,04727	0,11041	0,09597	0,46897	1,23562
GlycineMax	GM	1,66484	0,08316	1,64618	0,10884	0,08208	0,02812	0,22574	0,53525	0,06217	0,01930	0,00000	0,04010	0,02798	0,12961	0,11525	0,48825	1,25033
ManihotEsculenta	ME	1,69807	0,12311	1,67920	0,14889	0,12200	0,01494	0,26582	0,57534	0,10227	0,05934	0,04010	0,00000	0,01301	0,16971	0,15508	0,52831	1,27872
PopulusTrichocarpa	PT	1,68997	0,11113	1,67119	0,13673	0,11006	0,01135	0,25357	0,56289	0,08996	0,04727	0,02798	0,01301	0,00000	0,15737	0,14323	0,51611	1,27264
VitisVinifera	VV	1,55951	0,04770	1,54163	0,02167	0,04899	0,15689	0,09626	0,40568	0,06744	0,11041	0,12961	0,16971	0,15737	0,00000	0,02173	0,35880	1,16311
SelaginellaMoellendorffii	SM	1,56271	0,03221	1,54459	0,01114	0,03321	0,14155	0,11178	0,42175	0,05430	0,09597	0,11525	0,15508	0,14323	0,02173	0,00000	0,37362	1,16143
PhyscomitrellaPatens	PP	1,27644	0,40528	1,26143	0,37942	0,40642	0,51508	0,26255	0,05722	0,42615	0,46897	0,48825	0,52831	0,51611	0,35880	0,37362	0,00000	0,95335
ChlamydomonasReinhardtii	CR	0,50037	1,18731	0,47636	1,17236	1,18751	1,26427	1,09868	0,96503	1,20762	1,23562	1,25033	1,27872	1,27264	1,16311	1,16143	0,95335	0,00000

Table S20: Phylogenetic distance matrix based on **gene alignment**. Each pair represent the distance formed among each pair of the 17 plants species.

		BD	HV	OSJ	PV	SI	SB	ZM	AT	BRP	CP	GM	ME	PT	VV	SM	PP	CR
BrachypodiumDistachyon	BD	0,00000	0,03422	0,05974	0,05522	0,04918	0,05793	0,06173	0,38072	0,40739	0,32334	0,39652	0,33882	0,35533	0,30870	1,45847	0,78716	1,68287
HordeumVulgare	HV	0,03422	0,00000	0,05561	0,05109	0,04505	0,05379	0,05760	0,37658	0,40326	0,31921	0,39239	0,33469	0,35120	0,30457	1,45434	0,78303	1,67874
OryzaSativaJaponica	OSJ	0,05974	0,05561	0,00000	0,05048	0,04444	0,05318	0,05699	0,37597	0,40265	0,31860	0,39178	0,33408	0,35059	0,30396	1,45373	0,78241	1,67813
PanicumVirgatum	PV	0,05522	0,05109	0,05048	0,00000	0,00604	0,02084	0,02465	0,36438	0,39105	0,30700	0,38018	0,32248	0,33900	0,29236	1,44214	0,77082	1,66653
SetariaItalica	SI	0,04918	0,04505	0,04444	0,00604	0,00000	0,01480	0,01861	0,35834	0,38502	0,30097	0,37415	0,31645	0,33296	0,28633	1,43610	0,76478	1,66049
SorghumBicolor	SB	0,05793	0,05379	0,05318	0,02084	0,01480	0,00000	0,01131	0,36708	0,39376	0,30971	0,38289	0,32519	0,34170	0,29507	1,44484	0,77353	1,66924
ZeaMays	ZM	0,06173	0,05760	0,05699	0,02465	0,01861	0,01131	0,00000	0,37089	0,39757	0,31352	0,38670	0,32900	0,34551	0,29888	1,44865	0,77733	1,67305
ArabidopsisThalianaCol	AT	0,38072	0,37658	0,37597	0,36438	0,35834	0,36708	0,37089	0,00000	0,05195	0,10306	0,22400	0,16630	0,18282	0,15269	1,41591	0,74459	1,64030
BrassicaRapaPekinensis	BRP	0,40739	0,40326	0,40265	0,39105	0,38502	0,39376	0,39757	0,05195	0,00000	0,12973	0,25068	0,19298	0,20949	0,17937	1,44258	0,77126	1,66698
CaricaPapaya	CP	0,32334	0,31921	0,31860	0,30700	0,30097	0,30971	0,31352	0,10306	0,12973	0,00000	0,16662	0,10893	0,12544	0,09531	1,35853	0,68721	1,58292
GlycineMax	GM	0,39652	0,39239	0,39178	0,38018	0,37415	0,38289	0,38670	0,22400	0,25068	0,16662	0,00000	0,16893	0,18545	0,16849	1,43171	0,76039	1,65610
ManihotEsculenta	ME	0,33882	0,33469	0,33408	0,32248	0,31645	0,32519	0,32900	0,16630	0,19298	0,10893	0,16893	0,00000	0,08577	0,11079	1,37401	0,70269	1,59840
PopulusTrichocarpa	PT	0,35533	0,35120	0,35059	0,33900	0,33296	0,34170	0,34551	0,18282	0,20949	0,12544	0,18545	0,08577	0,00000	0,12731	1,39052	0,71921	1,61492
VitisVinifera	VV	0,30870	0,30457	0,30396	0,29236	0,28633	0,29507	0,29888	0,15269	0,17937	0,09531	0,16849	0,11079	0,12731	0,00000	1,34389	0,67257	1,56828
SelaginellaMoellendorffii	SM	1,45847	1,45434	1,45373	1,44214	1,43610	1,44484	1,44865	1,41591	1,44258	1,35853	1,43171	1,37401	1,39052	1,34389	0,00000	1,21767	2,11338
PhyscomitrellaPatens	PP	0,78716	0,78303	0,78241	0,77082	0,76478	0,77353	0,77733	0,74459	0,77126	0,68721	0,76039	0,70269	0,71921	0,67257	1,21767	0,00000	1,24391
ChlamydomonasReinhardtii	CR	1,68287	1,67874	1,67813	1,66653	1,66049	1,66924	1,67305	1,64030	1,66698	1,58292	1,65610	1,59840	1,61492	1,56828	2,11338	1,24391	0,00000

Supplementary Figures

Topological assessment of metabolic networks reveals evolutionary information

Jeaneth Machicao, Humberto A. Filho, Daniel J. G. Lahr, Marcos Buckeridge, and Odemir M. Bruno*
* Author for correspondence: bruno@ifsc.usp.br

Figure S21. Scatter plot of the two principal components of the topological networks measures, namely (a) hub-score, (b) degree, (c) in-degree, (d) out-degree, (e) local clustering coefficient, (f) autorithy-score, (g) local efficiency, (h) betweenness and (i) eigecentrality measure, using the the feature vector composed by the ***full-metabolites-set***, which contains the full content of metabolites among the 17 plants.

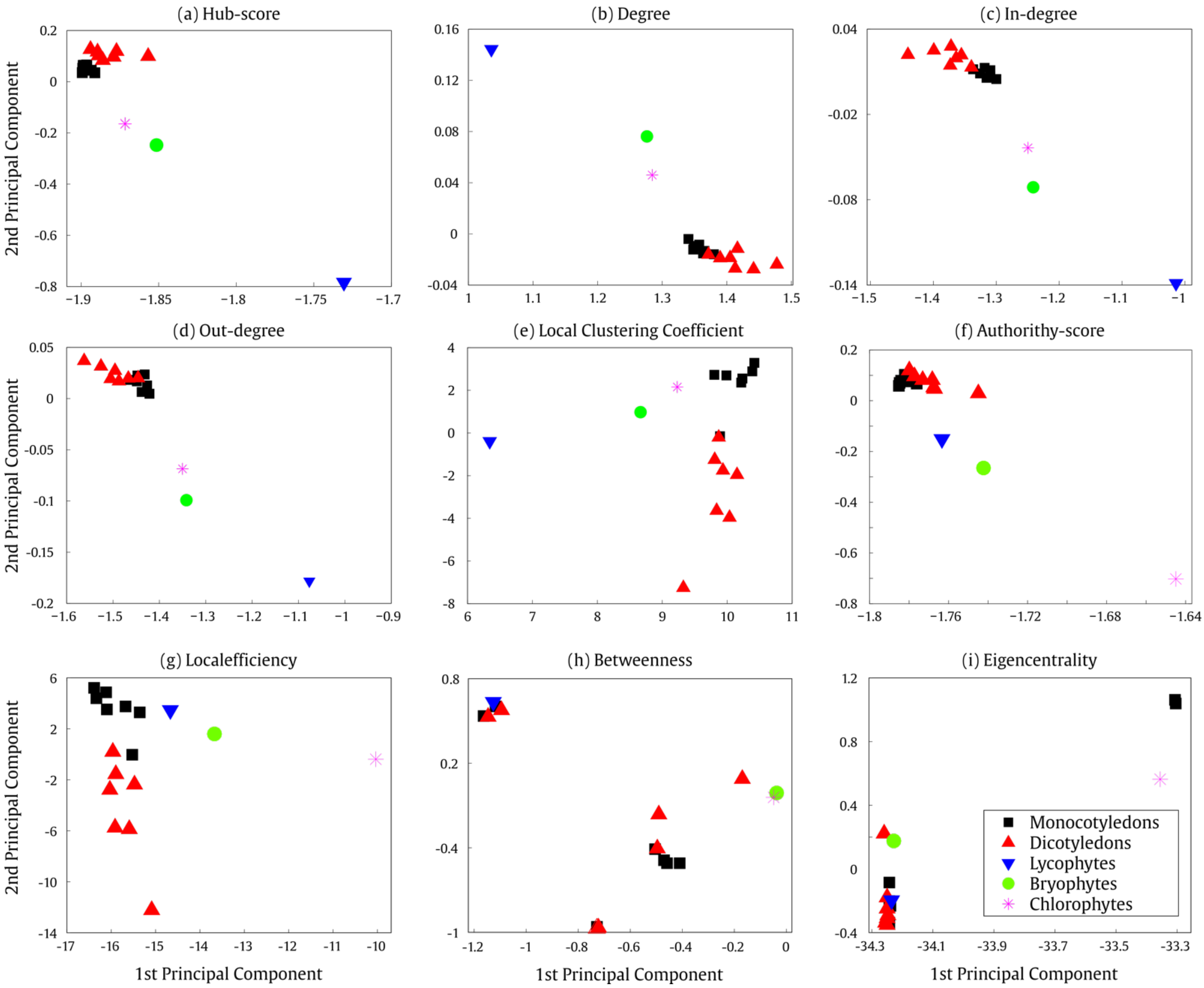

Figure S22. Clustergrams of 17 plants from PlantCyc metabolic reaction database, based on (a) hub-score, (b) degree metric, (c) in-degree metric, (d) out-degree, (e) local clustering coefficient (f) authority-score, (g) local efficiency, (h) betweenness and (i) eigecentrality measure, using the ***full-metabolites-set***, which contains the full content of metabolites among the 17 plants. The dash containing numbers (0.1, 0.05, and 1) shows the distance scale unit of the dendrogram. A scale bar (heatmap) showed at the right of each dendrogram indicates the clustering grouping.

(a) Clustergram tree based on hub-score measure (CPCC=0.89).

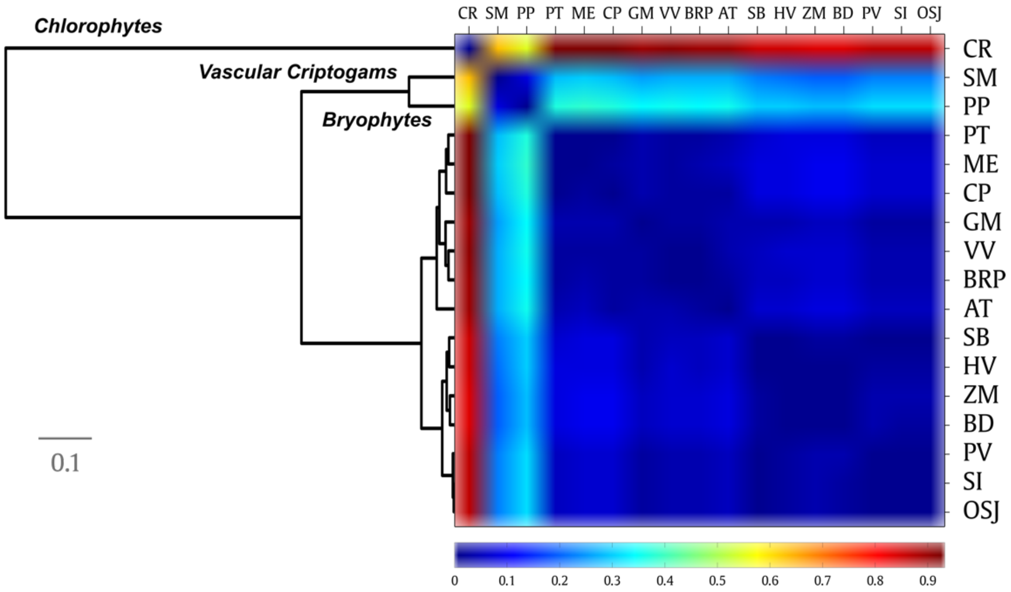

(b)Clustergram tree based on the degree (CPCC=0.86).

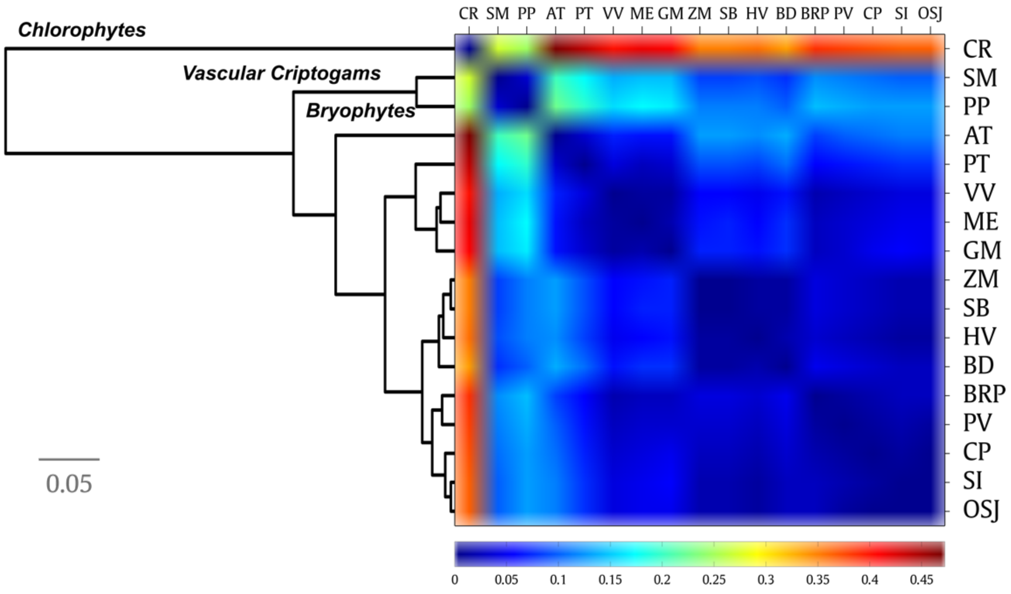

(c) Clustergram tree based on the in-degree measure (CPCC=0.86).

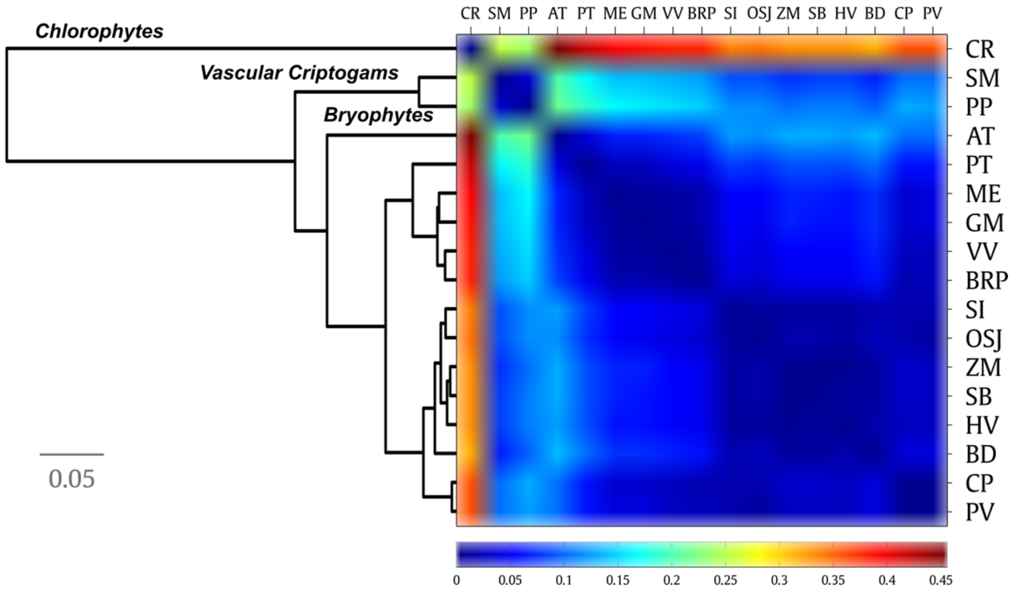

(d) Clustergram tree based on the out-degree measure (CPCC=0.88).

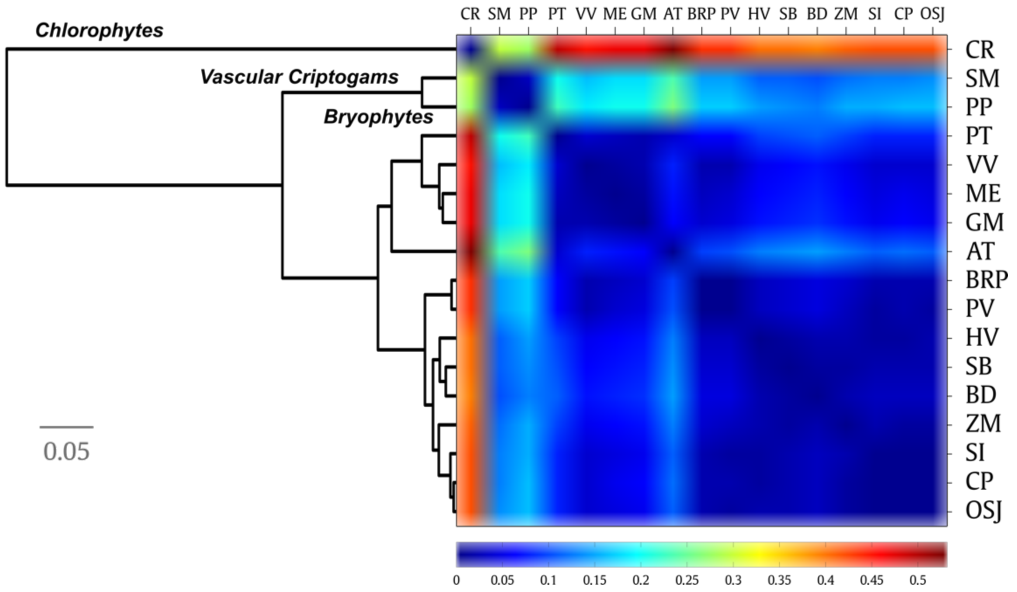

(e) Clustergram tree based on the local clustering coefficient measure (CPCC=0.14).

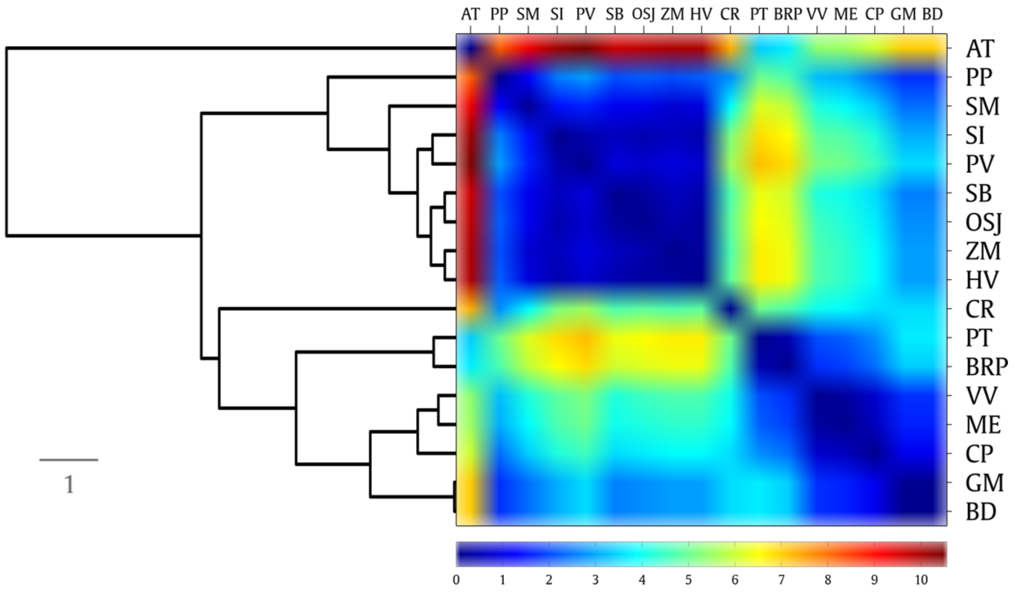

(f) Clustergram tree based on the authority-score measure (CPCC=0.72).

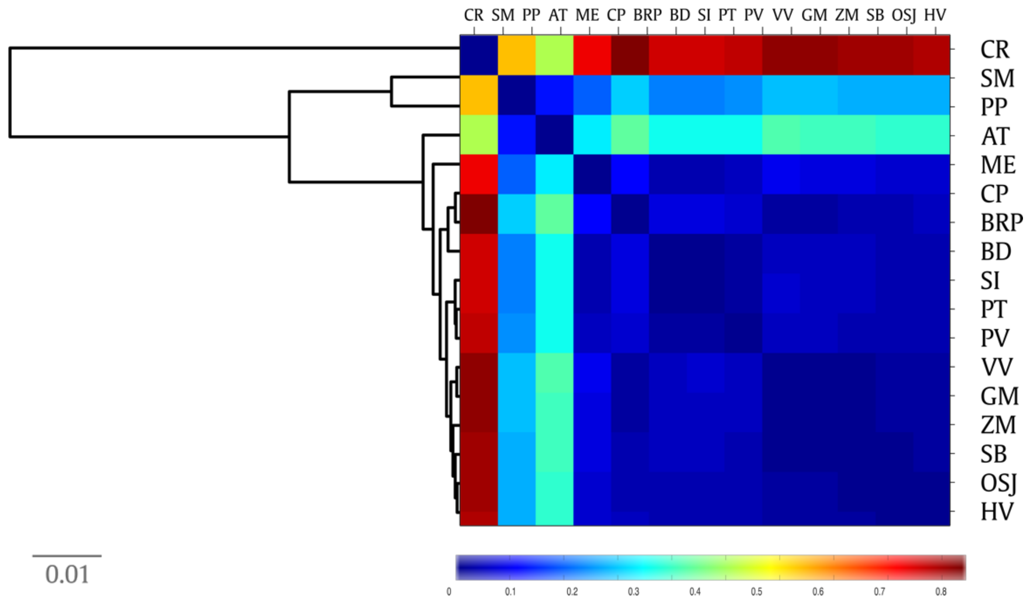

(g) Clustergram tree based on the local effectivity measure (CPCC=0.11).

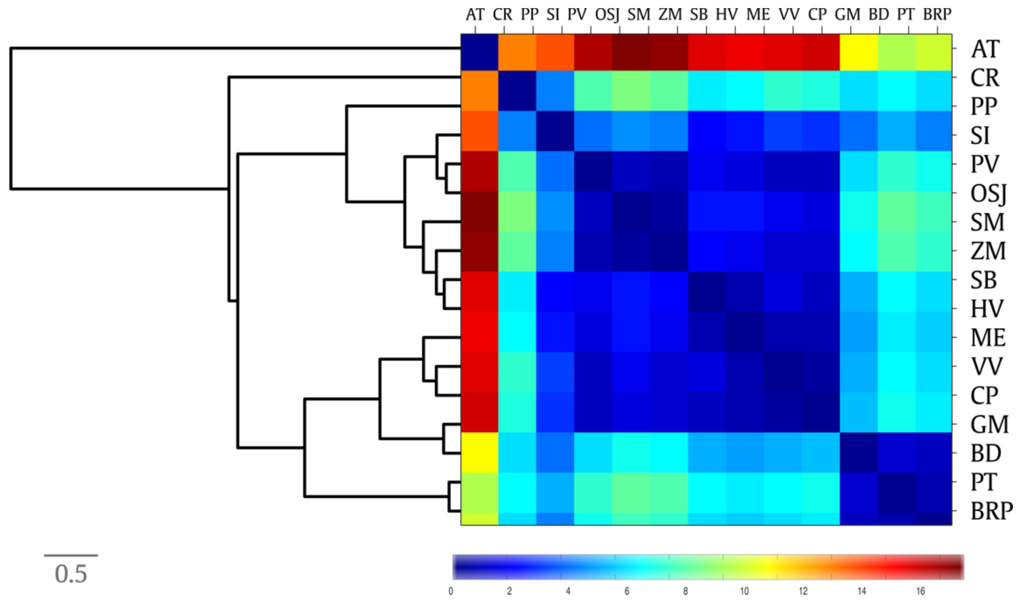

(h) Clustergram tree based on the betweenness measure (CPCC=0.03).

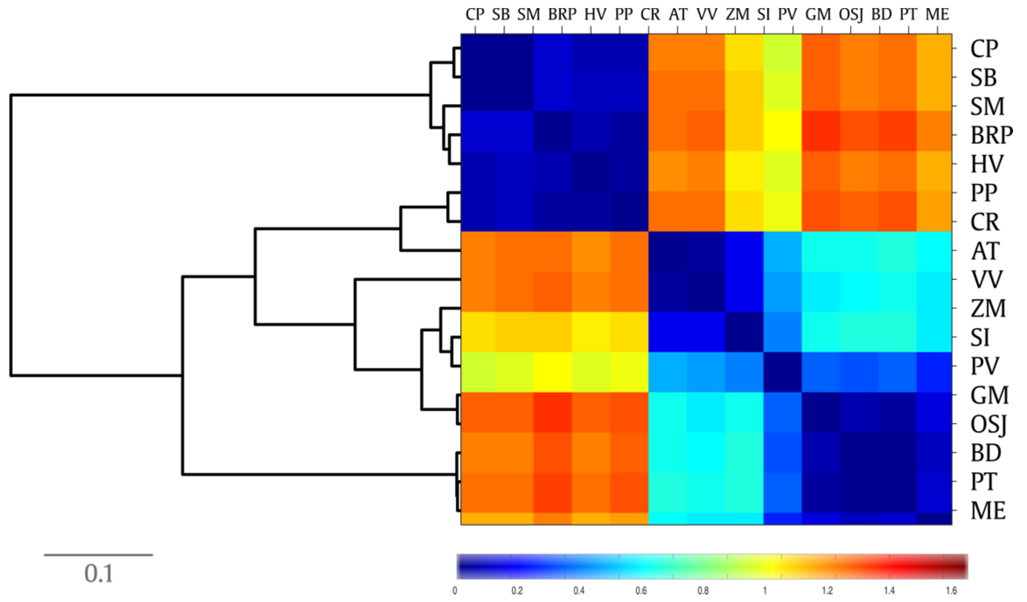

(h) Clustergram tree based on the eigencentrality measure (CPCC=0.27).

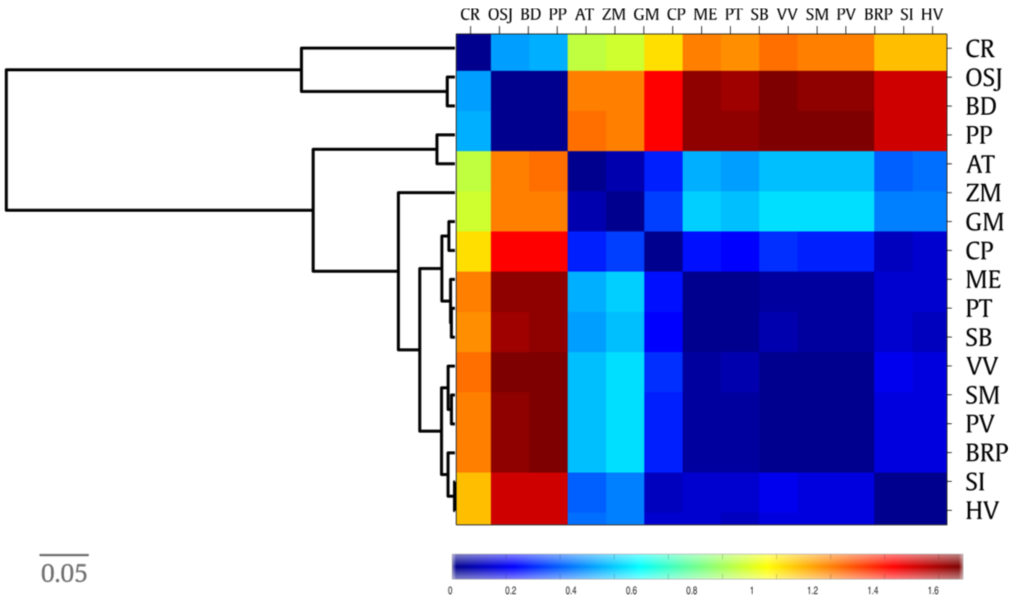

Supplement: Supplementary file 1 — Supplementary Information [file 41598_2018_34163_MOESM1_ESM.pdf]
